# Multispecies Outcomes of Sympatric Speciation after Admixture with the Source Population in Two Radiations of Nicaraguan Crater Lake Cichlids

**DOI:** 10.1371/journal.pgen.1006157

**Published:** 2016-06-30

**Authors:** Andreas F. Kautt, Gonzalo Machado-Schiaffino, Axel Meyer

**Affiliations:** Department of Biology, University of Konstanz, Konstanz, Baden-Württemberg, Germany; University of Wisconsin–Madison, UNITED STATES

## Abstract

The formation of species in the absence of geographic barriers (i.e. sympatric speciation) remains one of the most controversial topics in evolutionary biology. While theoretical models have shown that this most extreme case of primary divergence-with-gene-flow is possible, only a handful of accepted empirical examples exist. And even for the most convincing examples uncertainties remain; complex histories of isolation and secondary contact can make species falsely appear to have originated by sympatric speciation. This alternative scenario is notoriously difficult to rule out. Midas cichlids inhabiting small and remote crater lakes in Nicaragua are traditionally considered to be one of the best examples of sympatric speciation and lend themselves to test the different evolutionary scenarios that could lead to apparent sympatric speciation since the system is relatively small and the source populations known. Here we reconstruct the evolutionary history of two small-scale radiations of Midas cichlids inhabiting crater lakes Apoyo and Xiloá through a comprehensive genomic data set. We find no signs of differential admixture of any of the sympatric species in the respective radiations. Together with coalescent simulations of different demographic models our results support a scenario of speciation that was initiated in sympatry and does not result from secondary contact of already partly diverged populations. Furthermore, several species seem to have diverged simultaneously, making Midas cichlids an empirical example of multispecies outcomes of sympatric speciation. Importantly, however, the demographic models strongly support an admixture event from the source population into both crater lakes shortly before the onset of the radiations within the lakes. This opens the possibility that the formation of reproductive barriers involved in sympatric speciation was facilitated by genetic variants that evolved in a period of isolation between the initial founding population and the secondary migrants that came from the same source population. Thus, the exact mechanisms by which these species arose might be different from what had been thought before.

## Author Summary

Speciation is the main driver of biological diversity and how species arise is a central question in evolutionary biology. For speciation to occur in sexually reproducing organisms the exchange of genetic material (gene flow) between populations has to be reduced. Ultimately this has to be due to genetically determined reproductive incompatibilities between species. Yet, whether (an initial period of) geographic isolation is necessary for these incompatibilities to evolve has been subject to one of the most persistent debates in evolutionary biology. Sympatric speciation is the most extreme case of primary divergence-with-gene-flow and lies at the heart of this question. However, only few empirical examples of sympatric speciation are generally accepted and in most of these cases some ambiguities and doubts remain. This study provides evidence that the Nicaraguan crater lake cichlids can indeed be considered a valid example of sympatric speciation in the sense that the species themselves probably started to diverge in the absence of geographic barriers. However, the data also suggests that this divergence in sympatry may have been facilitated by genetic variants that evolved during a time of isolation between an initial founding population and a secondary wave of colonizers stemming from the same source population. This highlights the limitations in the definitions of sympatric speciation when the mosaic nature of genomes is taken into account: some of the genetic regions driving divergence may have evolved in allopatry while the populations themselves diverged in sympatry.

## Introduction

Understanding how populations can diverge and become distinct species in the presence of gene flow is a central objective in evolutionary biology [1–3]. That gene flow poses a problem for speciation has for long been known [4–6]. Gene flow and recombination homogenize the genomes of diverging populations and break down associations of loci relevant for ecological adaptations and assortative mating; a condition usually required for speciation [7–9]. Yet, a growing body of research has shown that speciation *can* progress in the presence of gene flow [2, 10–13]. Without a good understanding of the populations’ past it is, however, often difficult to distinguish between primary divergence-with-gene-flow and the sorting out of already partly diverged populations after secondary contact [3, 14]. This distinction is important as the latter involves a period of geographic isolation in which the abovementioned problem of gene flow and recombination does not arise [2, 15]. The evolution of reproductive incompatibilities in geographic isolation (allopatry) is well understood and not controversial, while primary divergence-with-gene-flow in the absence of strong geographic barriers demands other explanations [16]. From a population genetic perspective, the most extreme case of primary divergence-with-gene-flow is sympatric speciation [17]. In a biogeographic sense, sympatric speciation can be broadly defined as speciation in the complete absence of geographic (external) barriers [18]. The two definitions are not always in concordance [19–21], but the ultimate question that relates both and motivates the study of sympatric speciation is whether and to what extent speciation requires the mediating effects of a period of geographic isolation. In other words, is geographic isolation necessary to reduce gene flow and initiate population divergence in the first place or can speciation commence in a panmictic population? Thus, sympatric speciation has for long attracted theoreticians and empiricists alike, not because it is believed to occur frequently, but because—being the endpoint of the continuum of primary divergence-with-gene-flow—it may be particularly informative on the ecological conditions and evolutionary mechanisms that can lead to speciation in the presence of gene flow [19, 22, 23].

While theoretical models have shown that sympatric speciation is possible [8, 22, 24–26], only few convincing empirical case studies have been published [reviewed in ref. 1, 22]. And even in some of these cases critics remained doubtful [27, 28]. This is partly due to the fact that speciation with geographic isolation is generally considered much more plausible, almost like a null hypothesis in speciation. Sympatric speciation appears thus not only to be rare, but also hard to demonstrate empirically. In their seminal book Coyne and Orr [16] proposed four criteria that have to be fulfilled to demonstrate that sympatric speciation is the most likely mode of speciation: (i) sympatric distribution of contemporary species, (ii) genetically-based reproductive isolation, (iii) phylogenetic sister relationship, and (iv) no historic phase of geographic isolation. Several cases are in concordance with some of these criteria, but almost none unambiguously fit all four [18, 22]. Particularly the latter two criteria are inherently difficult to address and demonstrate. This is because a sister relationships between species (criterion iii) must reflect a true lineage bifurcation event and not simply result from a close genetic relationship due to secondary gene flow of evolutionarily more distantly related taxa. Especially inferences based on mitochondrial DNA alone are prone to error due to haplotype replacement [29–31], but nuclear markers can lead to false inferences too, if gene flow and incomplete lineage sorting are not accounted for [32, 33].

Further, demonstrating that a past allopatric phase of currently sympatrically occurring true sister species is unlikely (criterion iv), can be difficult to do in practice. Following [34] the problem is that essentially three different scenarios can be imagined that would be consistent with the first three but differ in the fourth of Coyne and Orr’s criteria for sympatric speciation: (1) sympatric speciation after a single colonization, (2) sympatric speciation after several colonizations from the same ancestral lineage and the putative formation of a hybrid swarm, and (3) speciation after secondary contact and introgressive hybridization. The first scenario can be considered the ‘purest’ form of sympatric speciation in which reproductive barriers arise completely in sympatry. In the second scenario some of the genetic variation later involved in reproductive isolation could have evolved in the time of separation of the primary founder population and the secondary migrants. Importantly, these genetic variants would not immediately lead to divergence, but be absorbed into the gene pool—potentially leading to a hybrid swarm—and only later be recruited in the speciation process [35, 36]. Speciation in this scenario could still be considered sympatric as population divergence happened in sympatry [34]; yet there is a role of geographic isolation if the admixture event was essential for speciation in sympatry. In the third scenario an initial level of (incomplete) divergence between the species evolved in geographic isolation, which would be strengthened by reinforcement [37] and/or ecological character displacement [38] upon secondary contact.

The first two scenarios predict equal levels of shared ancestry with outgroups and the source population and no signs of differential admixture (i.e. varying levels of admixture proportions) among sympatric species within a radiation, whereas the latter scenario of secondary contact predicts varying levels of shared outgroups ancestry and signs of differential admixture [34]. Distinguishing between these three scenarios is especially difficult if the source population is not known or extinct; an issue that leads to lingering doubts in even the otherwise most convincing cases of sympatric speciation [39]. The attainability of big genomic data sets as well as theoretical and methodological advances in recent years have, however, markedly increased the power to investigate more complex demographic scenarios of secondary gene flow, admixture, and multiple colonization events [40–42], thereby permitting to now infer if periods of geographical isolation were involved in putative cases of primary divergence-with-gene-flow and sympatric speciation. In this regard, recent evidence for a complex pattern of secondary gene flow and unequal shared outgroup ancestry of sympatric species of Cameroonian crater lake cichlids [34], has shed some new light on this traditionally considered prime example of sympatric speciation [43].

Crater lake cichlids in Nicaragua, belonging to the Midas cichlid species complex (*Amphilophus sp*.), represent a similar system in which fish from the two old and great lakes Managua and Nicaragua have repeatedly colonized small and isolated crater lakes [44]. The two great lakes are both inhabited by two species of Midas cichlids: *A*. *citrinellus* is a generalist species which presumably resembles the ancestral state and *A*. *labiatus* is adapted to feeding on invertebrates in rocky crevices with its characteristic hypertrophied lips and narrow head shape [45, 46]. While most crater lakes harbor only one (yet often polymorphic) population of Midas cichlids, in two of the crater lakes, Lake Apoyo and L. Xiloá, several endemic species have been described [44]. According to the current taxonomy Crater Lake Apoyo harbors six [47] and L. Xiloá four species of Midas cichlids [48]. The species differ in their ecology and, notably, in both crater lakes a species with an elongated body shape inhabiting the open water niche (from here on referred to as ‘limnetic’ as compared to the high-bodied and shore-associated ‘benthic’ species) has evolved independently [49]. The small size of the crater lakes, the fact that they are surrounded by steep crater walls and no water connections exists, and the complete endemism of Midas cichlid species suggested sympatric speciation to be the most parsimonious scenario. And indeed, genetic data supported the monophyly of Midas cichlids in L. Apoyo [50]. Yet, this first study was criticized because the different benthic species inhabiting L. Apoyo were not considered separately and only one of the species, *A*. *citrinellus*, from the source L. Nicaragua was considered in certain analyses [27]. Furthermore, the different species in L. Apoyo were not equidistant to the source population in genetic space as might be expected after sympatric speciation. Thus, according to the critics, the null hypothesis of multiple colonizations and introgressive hybridization could not be ruled out completely [27]. Later studies taking several or all six described species into account and using different genetic markers concluded sometimes in favor of monophyly of the L. Apoyo flock and thus sympatric speciation [49, 51, 52] and sometimes not [53]. In addition the assignment of individuals to the proposed six-species taxonomy did not match in many cases [49, 53]. Generally, L. Xiloá has been less in the focus of the debate around sympatric speciation, probably because its crater rim on the Eastern side is shallow and gene flow via intermittent direct water connections or vectors (e.g. birds) seems much more plausible than in the older, deeper and much more obviously isolated Crater Lake Apoyo. Nonetheless, also L. Xiloá’s species flock appears to be monophyletic [49, 52] and appears to have resulted from a single founder event [54]. But, a comprehensive investigation of the plausibility of sympatric speciation in L. Xiloá has never been done. In addition to the questions of monophyly and sympatric speciation there have been discrepancies in the inferred order of speciation events based on different markers and types of analyses [49, 52]. Most importantly, none of the abovementioned studies did explicitly take admixture between lakes, intralacustrine gene flow, and population size changes into account.

Nonetheless, Midas cichlids still feature as one of the most prominent examples of sympatric speciation [18]. In this study we use genome-level analyses and demographic modeling in a coalescent framework to reconstruct the evolutionary history of the two parallel radiations of Midas cichlids in L. Apoyo and L. Xiloá using a comprehensive RADseq data set. More specifically we address all major points of previous criticism and more recent doubts concerning sympatric speciation in Midas cichlids [27, 34]. To this end, we take all described species of Midas cichlids in the source and crater lakes into account and objectively assign individuals to genetic clusters to then (i) test for signs of unequal shared outgroup ancestry and differential admixture of sympatric species, (ii) establish the evolutionary relationships among species, and (iii) infer the demographic history of the two radiations to evaluate the evidence for primary divergence-with-gene-flow with or without secondary colonizations or secondary contact as outlined in the three scenarios of putative sympatric speciation above.

## Results

### Population structure

**Fig 1:**
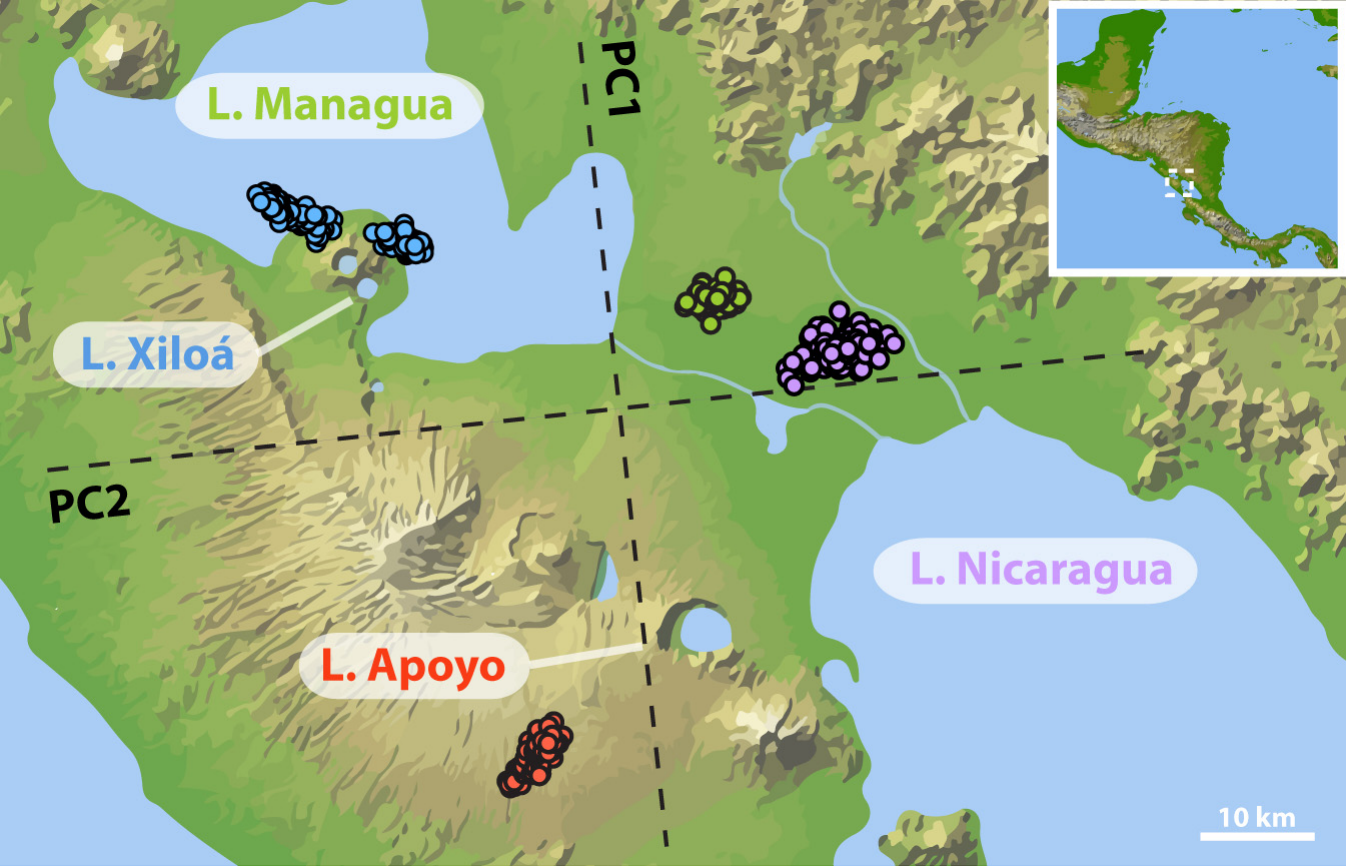
Lake populations form clearly distinct genetic clusters. Indicated on the geographic map are the locations of the two great lakes and the two focal crater lakes of this study. Superimposed are the first two main axes of genetic variation (principal components) based on 17,930 SNPs. PC1 and PC2 explain 3.81% and 2.08% of the overall genetic variation, respectively. Dots mark the position of individuals in two-dimensional genetic space and are color-coded by lake of origin. N = 123, 77, 124, 122 for lakes Xiloá, Managua, Apoyo, and Nicaragua, respectively.

Previous studies of Midas cichlids had been partially hampered by difficulties concerning the taxonomic classifications. Thus as a first objective we investigated the population structure in our comprehensive data set. We were interested in both signs of genetic exchange and relationships among lake populations as well as population structure and individual ancestry within crater lakes. To this end, using Principal Component Analyses (PCAs) [55] and *Admixture* [56], we first performed a ‘global’ analysis including all 446 individuals from the two great lakes and the crater lakes and then performed two ‘intralacustrine’ analyses focusing on each of the crater lakes separately. The first two principal components of the global PCA were highly significant (p-value ~ 0) and clearly separated the four lake populations (Fig 1). In concordance with the geographic proximity and the assumed colonization history, the genetic cluster of L. Apoyo was closer to L. Nicaragua and L. Xiloá was closer to L. Managua, while the two great lake populations were in close proximity in the two-dimensional genetic space. Interestingly, two distinct genetic clusters could be identified for L. Xiloá, one being slightly closer to L. Managua than the other one. Individuals in this cluster corresponded exclusively to the two species *A*. *amarillo* and *A*. *viridis*. This presumably closer affiliation of these two species to the source population was also apparent in the global *Admixture* analysis, albeit, and importantly, only when assuming *a priori* the same number of clusters as lakes (K = 4) (S1A Fig). Considering all lakes, the highest support was found for nine (K = 9) or twelve (K = 12) clusters; the cross-validation error was almost equally low for the two runs (S1B Fig). In the case of twelve clusters, four of the clusters corresponded to the two species *A*. *citrinellus* and *A*. *labiatus* in each L. Managua and L. Nicaragua while individuals from L. Xiloá and L. Apoyo were assigned to four different clusters each (S1A Fig). Notably, there were no signs of admixture between the lake populations anymore.

**Fig 2:**
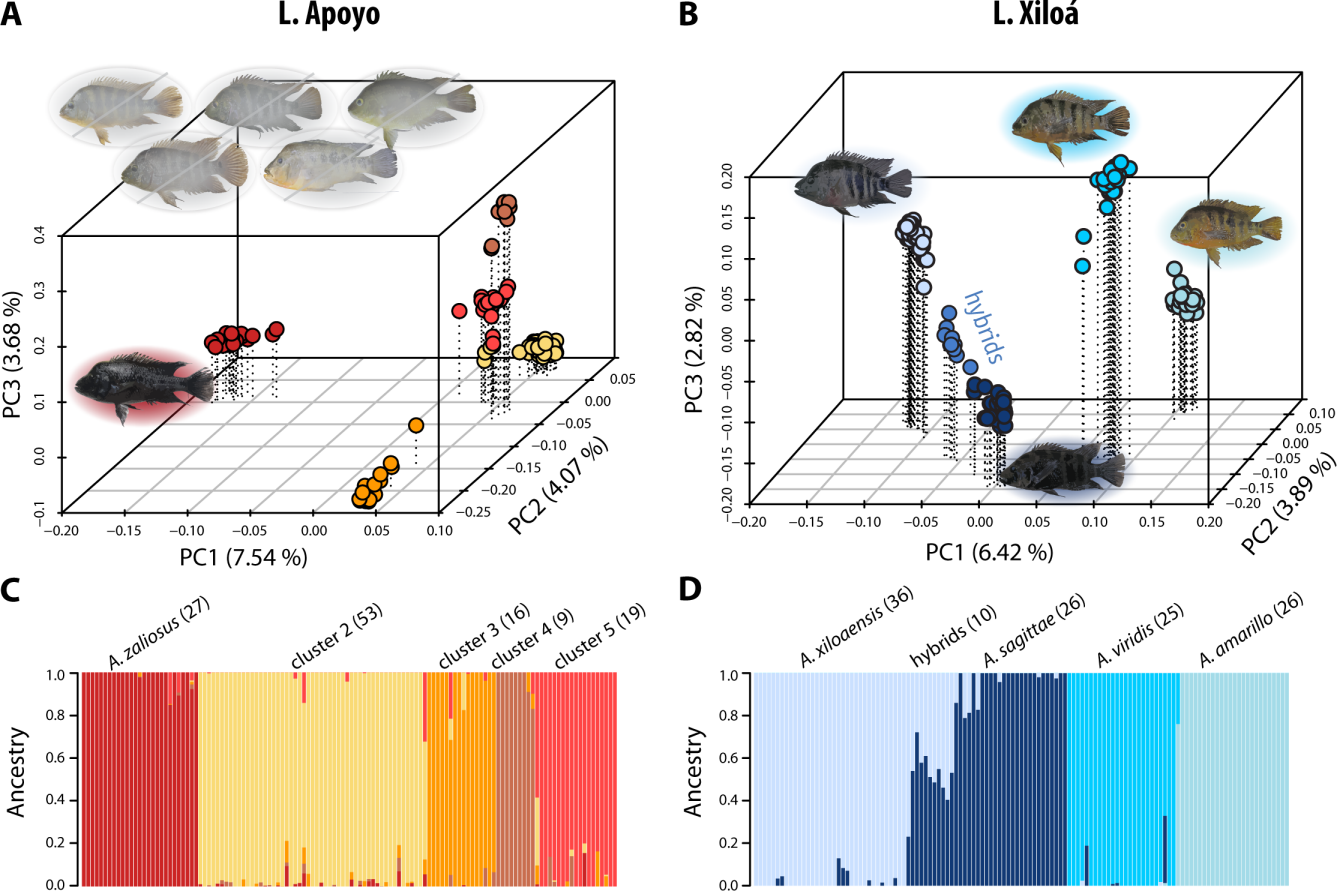
Sympatric species are genetically distinct, yet there is ongoing gene flow. Genetic clustering and individual ancestry of individuals within the two crater lakes both in form of A) B) the first three axes of genetic variation and C) D) the most supported number of clusters in an *Admixture* analysis (bottom panel). Analyses are based on 7,382 and 11,434 SNPs for L. Apoyo and L. Xiloá, respectively. Groups are labelled by species, if applicable, or genetic clusters as used in this study. Sample sizes are given in parentheses. Fish images next to species illustrate representative individuals. For L. Apoyo the five described benthic species are shaded in gray as they do not entirely match the genetic clusters.

In the intralacustrine *Admixture* analysis of L. Apoyo the occurrence of four and five clusters had the highest support (S2 Fig). Yet, 19 individuals, which are of strongly admixed ancestry in the case of four clusters (S1 Fig), formed a distinct cluster in the case of five clusters (Fig 2C). Five distinct clusters were also apparent in the PCA (Fig 2A). Thus, our set of samples from L. Apoyo seemed to be best described by five genetic clusters. The main axis of variation (PC1) clearly differentiated the limnetic *A*. *zaliosus* from the other four clusters. However, the delineation of the benthic individuals into the four different genetic clusters did in many cases not fit their species assignment based on morphology. Only in the case of *A*. *astorquii* were all individuals unambiguously assigned to one genetic cluster (cluster 2), albeit individuals from other species were included in this cluster as well. Since we think that the genetic clusters provide a more objective grouping of individuals than the sometimes difficult assignment based on morphology, we recoded benthic individuals as belonging to ‘clusters 2–5’ according to their genetic signature (S1 Table). Note that from here on we will essentially adopt a genetic cluster species concept [57] and use the terms species and cluster interchangeably. Furthermore, a few individuals from all genetic clusters exhibited signs of admixed ancestry.

In L. Xiloá three to four clusters had the highest support (S2 Fig) and four genetic clusters corresponded well to the four described species (Fig 2B). Only in seven out of 123 cases (three *A*. *amarillo* specimens assigned to *A*. *viridis* and four *A*. *sagittae* assigned to *A*. *xiloaensis*) did the species assignment not match (S1 Table) and individuals were re-assigned. However, the admixture plot also revealed a substantial amount of hybridization; eighteen individuals exhibited varying degrees of admixed ancestry between *A*. *sagittae* and *A*. *xiloaensis*, two *A*. *viridis* showed signs of admixture with *A*. *sagittae*, and one *A*. *amarillo* with *A*. *viridis*. The same pattern was also apparent in a plot of the first three eigenvectors of a PCA (Fig 2B). Putative hybrids are expected to occupy positions in genetic space along fictive lines connecting the species clusters [55]. Ten individuals in the center of this hybrid group, exhibiting more than 25% admixture proportions (on average 43%), were re-labeled as belonging to a ‘hybrid’ group and considered separately or excluded from all subsequent analyses. Our rationale for this was that the inclusion of such a number of obviously admixed individuals (also based on morphology, see below) might have had a strong impact on the phylogenetic and demographic analyses and in many cases it would have been difficult to decide to which species they should be assigned to. To further investigate the occurrence of hybridization within crater lakes we performed morphological analyses. Indeed, individuals in the hybrid group exhibited an intermediate morphology (S3 and S4 Figs). Thus, in both crater lake radiations we find evidence for distinct genetic clusters, yet also signs for ongoing gene flow. Pairwise levels of overall genetic differentiation among all species in the four lakes are provided in S2 Table. Patterns of genome-wide differentiation across the 24 linkage groups and among all sympatric species within the two crater lake radiations are visualized in S5 and S6 Figs. Analyses to detect loci putatively under divergent selection are described in S1 Text and detected outlier loci are given in S3 Table.

### Tests of differential admixture

The occurrence of two clusters in L. Xiloá in the global PCA, one being closer to L. Managua, would be consistent with two waves of colonization followed by introgressive hybridization. However, clustering methods do not explicitly take the demographic history into account and can thus sometimes falsely indicate admixture [58]. Thus we performed formal tests of admixture using *f3-statistics* [59]. *f3*-*statistics* are conceptually related to *D-statistics* (ABBA-BABA tests) and *f4*-*statistics* [60], and readily interpreted: a test population is compared to two reference populations and a significant negative value provides evidence that the test population experienced some form of admixture from populations related (or ancestral) to both reference populations. If the two species *A*. *amarillo* and *A*. *viridis*, which appear closer to L. Managua in the PCA—or more accurately their ancestral population–resulted from secondary contact and subsequent introgressive hybridization with the already established crater lake population, tests including one of these two species as a test population and one of the other two species from L. Xiloá together with a species from the source lake as reference populations may be expected to yield significant negative *f3-statistics*. Yet, none of the tests with this constellation returned a significant negative value (Table 1). In fact, we performed the test among all 1,092 possible three-population combinations (considering all populations and lakes in our data set) and only three tests returned a negative score, and none of those turned out to be significant. Thus, the *f3-statistics* do not provide evidence for secondary contact followed by introgressive hybridization. We note, however, that a history of admixture will not always result in negative *f3-statistics*, especially if the test population has experienced a lot of population-specific drift [60, 61]. We further note that tests based on the *f3-statistics* would not be able to detect an admixture event (secondary colonization) that occurred before the sympatric species diverged as the test and reference populations of the crater lakes would share equal proportions of admixed genotypes.

**Table 1:** *f3-statistics* do not provide evidence for secondary contact and introgression.

Test; Reference1, Reference2 L. Xiloá; L. Xiloá, L. Managua	f3-statistic	Standard error	Z-score
*A*. *amarillo; A*. *sagittae*, *A*. *citrinellus*	0.002264	0.000254	8.918
*A*. *amarillo; A*. *sagittae*, *A*. *labiatus*	0.002152	0.000266	8.078
*A*. *amarillo; A*. *xiloaensis*, *A*. *citrinellus*	0.002587	0.000275	9.391
*A*. *amarillo; A*. *xiloaensis*, *A*. *labiatus*	0.002543	0.000291	8.726
*A*. *viridis; A*. *sagittae*, *A*. *citrinellus*	0.000005	0.000195	0.247
*A*. *viridis; A*. *sagittae*, *A*. *labiatus*	0.000005	0.000219	0.022
*A*. *viridis; A*. *xiloaensis*, *A*. *citrinellus*	0.001951	0.000276	7.079
*A*. *viridis; A*. *xiloaensis*, *A*. *labiatus*	0.001976	0.000291	6.799

Significant *negative* values of the *f3-statisic* would provide evidence for admixture of the Test population. Shown is a subset of tests involving the two species in L. Xiloá that showed a closer position to the source population in L. Managua in the global PCA as a test population together with any of the other two species in L. Xiloá from the more distant cluster and any of the two species in the source lake L. Managua as reference populations. None of the 1,092 performed tests among different lakes and populations was significant.

Another way to investigate possible admixture events is by placing migration edges on a phylogenetic tree and evaluating whether they improve the fit of the model (tree) by reducing deviations in the residual covariance matrix: positive residuals indicate populations that exhibit observed covariances that are higher than accounted for by the model [61]. We used *Treemix* to build a tree and placed up to four migration edges (m) on it. The tree without migration (m = 0) provided already a relatively good fit to our data: the fraction of variance explained in the observed covariance matrix by the tree (“f” according to [61]) was 99.7%. Importantly, no stark positive residual covariances between any of the source populations and any of the crater lake species was apparent (S7 Fig). Adding four migration edges (m = 4) improved the fit of the tree slightly (f = 99.9%). The first three putative migration edges were placed *between* sympatric species *within* the two crater lakes (S7C, S7E and S7G Fig) and the fourth one between the ancestor of all L. Xiloá species and *A*. *viridis* from L. Xiloá itself (S7I Fig). The latter migration edge is difficult to interpret as we would expect secondary gene flow from the source population or a related species into a crater lake species to be reflected by a migration edge coming from the lineage leading to the two species in the respective source lake, but not from its own ancestral lineage. We note that we are not aware of any closely related species that could have hybridized with a Midas cichlid species in the last few thousand years. Furthermore, the small increase in fit provided by the fourth migration edge does not come from a decrease of positive residual covariances between *A*. *viridis* (or any other species in L. Xiloá) and the source populations—the fit is already good without any migration (S7B Fig). Instead, it seems to improve the fit of the relationships among species within L. Xiloá (S7H and S7J Fig). Thus, rather than indicating secondary gene flow from the source (or a related) population into *A*. *viridis* we think this migration edge rather reflects the difficulty of fitting the evolutionary relationships of the species within L. Xiloá in a bifurcating tree (even with migration): the topology within L. Xiloá is not robust and when fitting three migration edges *A*. *viridis*, and not *A*. *amarillo*, is the first species to split (S7G Fig). That some of the divergence events do not adhere to a strict bifurcating manner was also supported by other phylogenetic analyses that we performed (see below).

In any case, the *f3-statistics* did not provide evidence for differential admixture of *A*. *viridis* and this putative migration edge is thus not significant: the statistical support of migration edges in *Treemix* have to be considered with caution and three- or four-population tests are recommended as formal tests of admixture [61]. We stress that we used *Treemix* in an explorative approach, but refer readers to the *f3-statistics* for formal tests of differential admixture. Given the high fit of the model with four migration edges and the fact that the highest scaled residual covariance between any two populations was very low with less than 1.5 SE, we did not attempt to fit more than four migration edges.

Monophyly of the two radiations was strongly supported (100% bootstrap support). Also the phylogenetic sister relationship of L. Managua and L. Xiloá, providing evidence for the former being the source of the latter, was found in a 100% of bootstrap replicates. Interestingly, apart from the node grouping *A*. *sagittae*, *A*. *xiloaensis*, and the hybrid group in L. Xiloá (100% bootstrap support), the branching order within the radiations was not well supported (bootstrap support ranged from 59.8%–86.2%).

### Phylogenetic analyses

**Fig 3:**
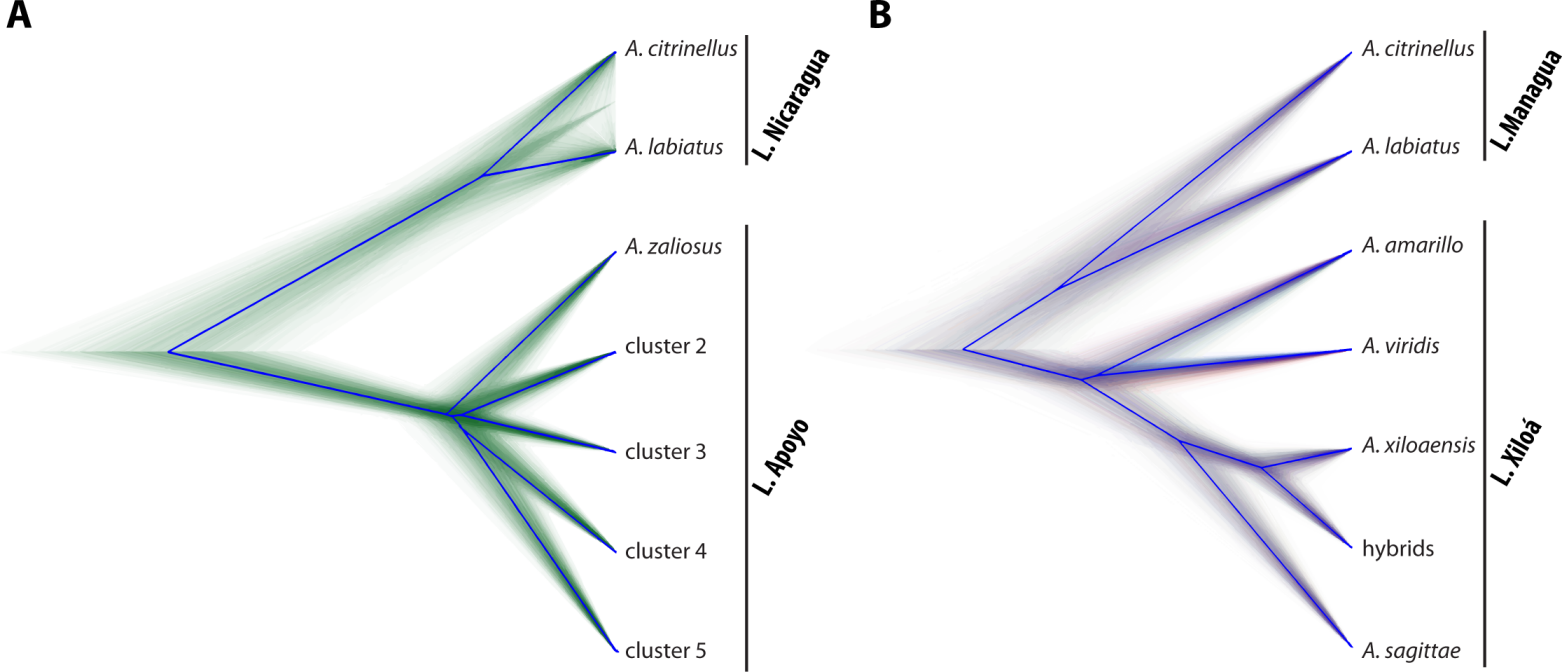
Multispecies outcomes of sympatric speciation. Cloudograms of the radiation of A) L. Apoyo and B) L. Xiloá with the two species from their respective source lake. Trees were obtained with *SNAPP* and are based on four randomly selected individuals per species and SNP matrices without missing data (1,772 and 1,290 SNPs for L. Apoyo and L. Xiloá, respectively). Thin lines represent individual trees and thick blue lines indicate the overall supported topology (root canal).

The low bootstrap support of nodes *within* the crater lake radiations in our *Treemix* tree led us to further investigate the evolutionary relationships among the sympatric species. To this end, we first built phylogenetic trees using *SNAPP*, which is explicitly designed to handle biallelic markers such as SNPs and employs the multispecies coalescent [62]. *SNAPP* returns a sample of species trees, which can be visualized in a “cloudogram”. Due to the computational burden and since we were only interested in the topology as well as relative branching times *within* the two radiations we built two separate trees, as their respective monophyly was strongly supported. In both trees it was evident that the two species from the source lakes are sister species and are equally distantly related to the crater lakes radiations (Fig 3). Within L. Apoyo the cloudogram indicated an almost starlike topology with extremely short internal nodes (Fig 3A). The overall consensus (root canal) suggested that *A*. *zaliosus* diverged first followed by a split of cluster 2–3 from cluster 4–5. Yet, every possible topology within the radiation was represented by some trees. In total 196 different consensus trees were found (differing in topology and divergence time). Also for the species that are endemic to L. Xiloá the cloudogram indicated a simultaneous split of three species (Fig 3B). *A*. *amarillo*, *A*. *viridis* and the ancestral population of *A*. *sagittae* and *A*. *xiloaensis* seemed to have split at the same time followed by the split of the latter two. Interestingly the hybrid group did not take an intermediate position between *A*. *sagittae* and *A*. *xiloaensis*, but formed the sister group to *A*. *xiloaensis* in all trees. The first three consensus trees (nine in total) covered 35%, 30%, and 27% of the individual trees and supported either a sister relationship of *A*. *amarillo* and *A*. *viridis*, an earlier split of *A*. *amarillo*, or an earlier split of *A*. *viridis*, respectively.

Due to the computational demand of this method the phylogenetic trees were limited to only four individuals per species and a subset of loci [63, 64]. To evaluate whether the phylogenetic results might be influenced by using only few individuals and excluding missing data [65], we built individual-based phylogenetic split networks including all samples and more markers allowing for missing data (see Methods for details). For both radiations they revealed essentially an identical pattern (S8 Fig). In L. Apoyo all species seemed to diverge simultaneously, whereas in L. Xiloá there was one split between *A*. *amarillo*, *A*. *viridis*, and the ancestor of *A*. *sagittae* and *A*. *xiloaensis*. The hybrid group occupied an intermediate position between the latter two species, which is expected considering that the networks were based on genetic distance. In both analyses the two species in the source lakes were almost not distinguishable and were equally distantly related to the crater lake radiations. The fact that the great lake species were almost not distinguishable is probably due to the fact that the networks were based on genetic distance only. Overall genetic differentiation between the great lake species was very low (S2 Table)—presumably due to their relatively large effective population sizes—leading to a low resolution in the networks. In the *SNAPP* analyses differences in effective population sizes were taken into account and the two species appeared probably therefore clearly diverged in the *SNAPP* trees in contrast to the networks.

### Demographic model selection

A limitation of the described phylogenetic methods is that they do not take gene flow and changing population sizes into account. Moreover, the *f3-statistics* may not detect admixture events that happened before the split of the sympatric species. To overcome these limitations and furthermore infer the demographic history of the radiations we used *fastsimcoal2* to perform coalescent simulations in pre-defined models and evaluated their fit against our empirical data summarized in the multi-dimensional site frequency spectrum (SFS) [66, 67]. To better account for the complexity of multi-population models, we started with one-population models for both species in both great lakes (the source populations). For each of the four populations six different models were tested (S9A Fig). A model incorporating a sudden reduction in population size in the past followed by exponential growth until the present (‘bottlegrowth’) had the highest support in all four populations (S4 Table). Since a signal of recent population expansion could be driven by rare alleles resulting from sequencing and genotyping error we repeated the analyses for *A*. *citrinellus* from Nicaragua using only genotype calls that were based on at least 15x coverage. Importantly, the ‘bottelgrowth’ model was again the most supported one (S4 Table).

Next, we tested each crater lakes species together with *A*. *citrinellus* from the respective great lake as a source population in two-population models (S9B Fig). We used *A*. *citrinellus* because it presumably resembles the ancestral state of fish in this species complex and because fish with hypertrophied lips, resembling *A*. *labiatus*, are not present in L. Apoyo and are extremely rare in L. Xiloá [44]. Moreover, our phylogenetic analyses suggest that both species in the source lakes are equally distantly related to the crater lake radiations (Fig 3). We tested between nine and eleven models for each species and the same class of model (differing only in migration) was supported for all species (S5 Table). This model included (i) exponential growth after a population bottleneck in the source population (‘bottlegrowth’), (ii) divergence followed by (iii) exponential growth in the crater lake species, and (iv) an admixture event from the source into the crater lake. In the case of L. Apoyo gene flow between the lakes was not supported, whereas in L. Xiloá migration from the source improved the fit of the model. However, the relative statistical support for the different models with or without migration is not very different (S5 Table). Thus, our data strongly support the population size changes and the admixture event, but we have rather low power to distinguish the different migration scenarios.

For all species a model in which the colonization event happened *after* (in forward time) the bottleneck in the source populations was superior to a model in which we forced the colonization to happen *before* the bottleneck (S5 Table). This could indicate a limitation of the inference method, as a bottleneck in the source could lead to a loss of information and bias lineages to coalesce before (backwards in time) the bottleneck [68]. In order to test this, we simulated data using the maximum likelihood parameter estimates and data structure of cluster 2 in L. Apoyo (i.e. using the same number and length of loci) but added 10,000 generations to the divergence time. Importantly, we were able to infer the correct (i.e. simulated) divergence time in this case (S6 Table). This suggests that theoretically we have enough power to correctly infer divergence times that happened before the bottleneck in the source populations.

**Fig 4:**
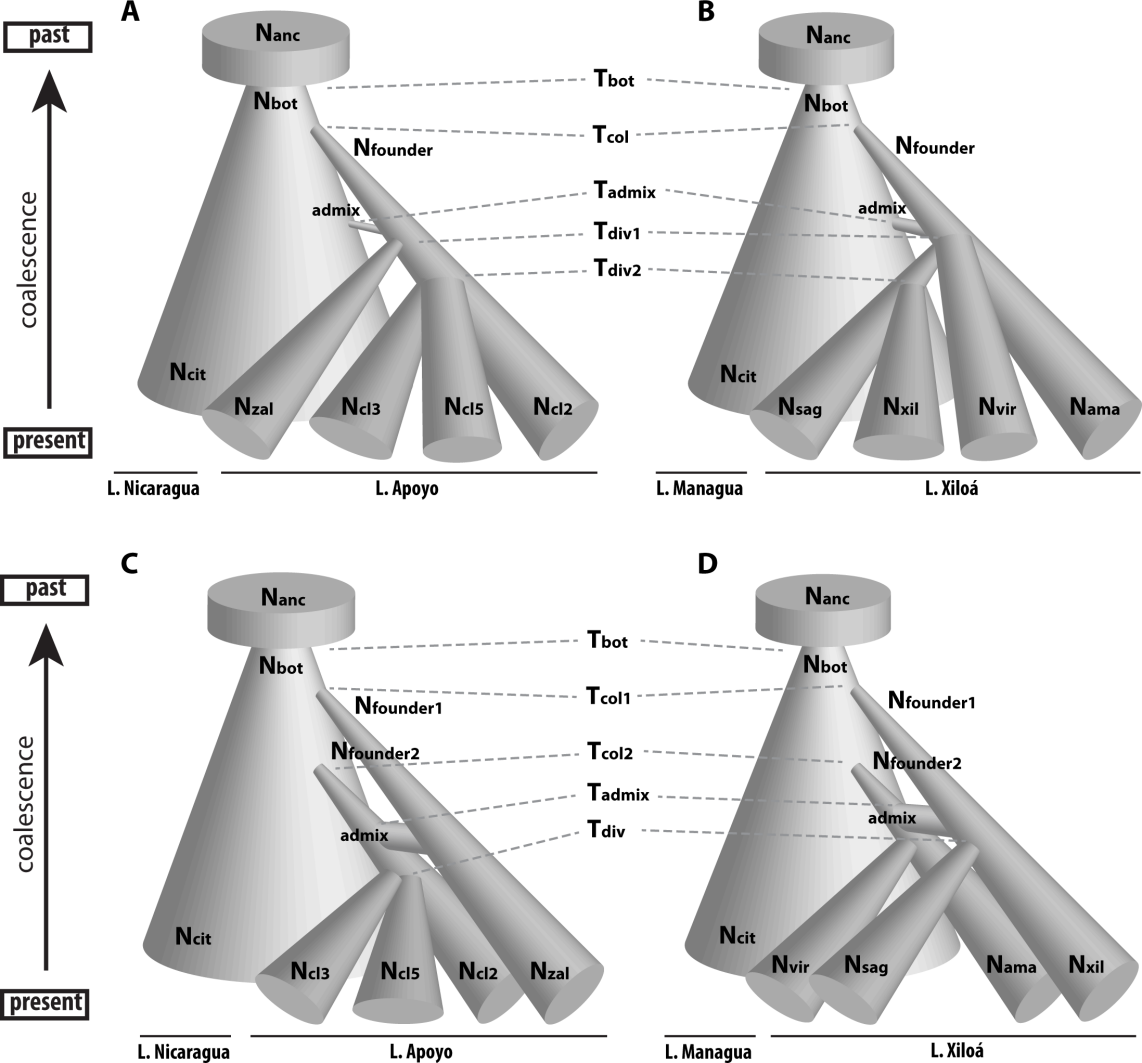
Demographic history of sympatric speciation. Schematic illustrations of the most supported demographic models of A) B) sympatric speciation and C) D) the alternative hypotheses of secondary contact for both radiations. Parameter estimates are provided in Table 3. Species names are abbreviated by their first three letters. Note that migration between sympatric species and from L. Managua into L. Xiloá was included in the model, but is not indicated. Furthermore, growth was modelled to be exponential and not linear as depicted here. Models are not drawn to scale but merely indicate relative differences.

Finally we analyzed the demographic history in five-population models. Even though the *f3-statistics* did not provide evidence for secondary contact we wanted to make use of the likelihood framework to explicitly evaluate the evidence for the two main competing hypotheses: sympatric speciation (after admixture from the source population) and secondary contact followed by introgressive hybridization (Fig 4). In addition, we aimed to evaluate different topologies within the radiations to further investigate the support for simultaneous divergence events. Building up on the results of the two-population models, for all models in both radiations we included a ‘bottlegrowth’ event in the source population and exponential growth in the crater lake species. Furthermore, in L. Apoyo we did not include gene flow between the lakes, whereas in models of L. Xiloá we allowed for migration from the source population (L. Managua) into the crater lake species. Migration was assumed to be identical, that is only one migration parameter was used. In both radiations we added gene flow between the sympatric species, assuming it again to be identical and symmetrical. While this assumption may be overly simplistic, including different migration parameters for all twelve possible migration routes would have likely over-parameterized our models.

For L. Apoyo we tested six different models. Five models of sympatric speciation and one model of secondary contact were evaluated. Within the sympatric speciation models our aim was to evaluate the support for three different topologies (see below), intralacustrine gene flow, and an admixture event prior to sympatric speciation (we refer to the model of sympatric speciation without prior admixture as “single colonization”). Incorporating an initial split of *A*. *zaliosu*s from the benthic species was strongly supported over a simultaneous split of all species (Table 2). However, including another parameter to model an additional split of cluster 5 from the other two benthic species, as weakly indicated in our phylogenetic analysis, did not increase the likelihood. Removing gene flow among the sympatric species or the admixture event into the crater lake population before sympatric speciation strongly decreased the likelihood of the model. With four species a multitude of two-colonization scenarios is conceivable, yet it is computationally unfeasible and biologically not sensible to test all possible models [69]. Hence, based on the firmly established finding that *A*. *zaliosus* is genetically the most distinct species within the radiation of L. Apoyo we formalized the main competing hypothesis of secondary contact as: an initial colonization by *A*. *zaliosus* followed by a secondary colonization by the ancestral population of the benthic species and admixture. This model was 2.5 times less likely than the best model of admixture prior to sympatric speciation.

**Table 2:** Support for five-population demographic models.

	Model	# parameters	Ln-Lhood	ΔAIC	Rel. Lhood
L. Apoyo	Admixture prior to sympatric speciation (“Best”)	18	-78856.161	-	-
	Secondary contact	18	-78857.096	1.870	0.393
	Best with additional split	19	-78856.175	2.028	0.363
	Best without initial split (all simultaneous)	17	-78860.518	6.713	0.035
	Best without intralacustrine migration	17	-78861.966	9.609	0.008
	Best without admixture (single colonization)	16	-78887.350	58.377	~0
L. Xiloá	Admixture prior to sympatric speciation (“Best”)	19	-95203.363	-	-
	Best with additional split *A*. *viridis*	20	-95202.951	1.176	0.556
	Best with additional split *A*. *amarillo*	20	-95203.423	2.120	0.347
	Best with additional split *A*. *amarillo+A*. *viridis*	20	-95203.598	2.470	0.291
	Best without additional split (all simultaneous)	18	-95205.627	2.527	0.283
	Secondary contact	19	-95207.623	8.520	0.014
	Secondary contact with two intralacustrine splits	20	-95207.510	10.293	0.006
	Best without intralacustrine migration	18	-95219.078	29.430	~0
	Best without admixture (single colonization)	17	-95232.772	54.817	~0

Given are the number of parameters, the Ln-Likelihood, the delta AIC (Akaike Information Criterion) value (all positive), and the relative likelihood for every model. The latter two are given in reference to the best model for each lake radiation. Models are ordered by decreasing support. The best model of sympatric speciation and the alternative model of secondary contact for each radiation are visualized in Fig 4. Other models in the list are denoted by addition or removal of parameters in reference to the best model. See also main text for details. For each model 125 independent *fastsimcoal2* runs were performed. The site frequency spectrum for L. Apoyo was built from 8,348 segregating and 3,233,075 total sites and for L. Xiloá from 9,416 and 4,142,231 total sites. Maximum Ln-Likelihood values given the observed SFS were -78840.296 and -95175.566 for L. Apoyo and L. Xiloá, respectively. The radiation of L. Apoyo was analyzed together with *A citrinellus* from L. Nicaragua, and L. Xiloá with *A*. *citrinellus* from L. Managua.

Similar to L. Apoyo, for L. Xiloá we tested seven models of sympatric speciation and two models of secondary contact. Modeling two intralacustrine divergence events, one between *A*. *amarillo*, *A*. *viridis*, and a third population which later split into *A*. *sagittae* and *A*. *xiloaensis*, was strongly supported over a model in which one ancestral population split simultaneously into all species. However, a sister relationships of *A*. *amarillo* and *A*. *viridis*, or an initial split by either of the two species did not further improve the model. Gene flow between the sympatric species and an admixture event before the onset of the radiation was again strongly supported (Table 2). Given the seemingly closer genetic affiliation of *A*. *amarillo* and *A*. *viridis* to the source population in our global PCA, we framed the main alternative hypothesis of secondary contact (in contrast to sympatric speciation) for L. Xiloá to be: an initial colonization by the ancestral population of *A*. *sagittae* and *A*. *xiloaensis* followed by a secondary colonization by the ancestral population of *A*. *amarillo* and *A*. *viridis* and subsequent admixture. For this type of model we tried two different topologies, one in which *A*. *amarillo* and *A*. *viridis* split before *A*. *sagittae* and *A*. *xiloaensis*, and a simultaneous split of the two lineages. The latter model was more strongly supported, yet it was about 70 times less likely than the model of sympatric speciation.

### Colonization history

#### Crater Lake Apoyo

According to the maximum likelihood point estimates of the most supported model (admixture prior to sympatric speciation) of L. Apoyo (Table 3; see also for 95% confidence intervals for all parameters in the best models) the source population in L. Nicaragua decreased from ca. 19,900 individuals to only ca. 4,200 individuals around 1,920 generations ago and has since been growing again to a current size of about 764,600 individuals. L. Apoyo was colonized by only approximately 260 fish around 1,690 generations ago and about 890 generations ago its population received approximately 4% of its gene pool from a second wave of colonization (i.e. an admixture event) from the source population. Immediately afterwards *A*. *zaliosus* diverged from the ancestral lineage of the other species, which split into the three benthic species about 680 generations ago. The current population sizes of the four sympatric species range from about 6,460 to 43,960 individuals, with *A*. *zaliosus* being the smallest. Migration (i.e. gene flow) between the sympatric species has happened with a probability equal to 7.5 every 100,000 alleles (7.5x10^-5^). Note that migration rates cannot be readily converted to number of individuals in growing populations, as the number of migrants is the product of the respective migration rate and the size of the receiving population, which is changing exponentially.

**Table 3:** Inferred parameters under models of admixture prior to sympatric speciation and secondary contact as defined in Fig 4.

L. Apoyo				L. Xiloá			
	Admixture prior to sympatric speciation		Secondary contact		Admixture prior to sympatric speciation		Secondary contact
Parameter	Point estimate	95% CI	Point estimate	Parameter	Point estimate	95% CI	Point estimate
Nanc	19,937	17,255–22,011	20,021	Nanc	18,322	15,232–20,045	18,362
Nbot	4,178	3,094–11,874	6,736	Nbot	1,846	1,392–6,410	1,750
Nfounder	263	128–738	293	Nfounder	146	37–557	225
Nfounder2			273	Nfounder2			665
Ncit	764,626	150,805–930,091	220,339	Ncit	287,268	107,605–869,793	373,192
Nzal	6,461	0–42,186	3,520	Nama	34,439	0–36,247	44,498
Ncl2	14,990	0–26,482	32,921	Nvir	35,544	13,767–49,894	35,867
Ncl3	38,411	153–44,161	21,773	Nsag	14,779	0–39,315	7,922
Ncl5	43,960	13,009–48,938	46,084	Nxil	12,144	252–43,605	6,291
Tbot	1,924	1,442–2,562	1,955	Tbot	1,421	1,390–2,344	1,252
Tcol	1,687	1,234–2,257	1,654	Tcol	1,318	1,198–2,064	1,135
Tcol2			1,154	Tcol2			869
Tadmix	892	859–1,568	907	Tadmix	891	767–1,374	850
Tdiv1	889	683–1,252	740	Tdiv1	876	706–1,197	825
Tdiv2	678	545–1,012		Tdiv2	752	613–1,043	
admix	0.043	0.009–0.093	0.896	admix	0.286	0.107–0.433	0.729
Mintra^a^	7.54 x 10^−5^	0–9.52 x 10^−5^	7.48 x 10^−5^	Mintra^a^	8.81 x 10^−5^	0–7.77 x 10^−5^	7.52 x 10^−5^
				MintoXil^b^	1.72 x 10^−5^	0–8.60 x 10^−5^	3.32 x 10^−5^
Rcit	2.71 x 10^−3^	1.32 x 10^−3^–3.02 x 10^−3^	1.78 x 10^−3^	Rcit	3.55 x 10^−3^	1.61 x 10^−3^–3.62 x 10^−3^	4.28 x 10^−3^
Rzal	3.52 x 10^−3^	1.70 x 10^−3^–7.25 x 10^−3^	1.50 x 10^−3^	Rama	4.15 x 10^−3^	1.01 x 10^−3^–4.09 x 10^−3^	4.84 x 10^−3^
Rcl2	2.40 x 10^−3^	4.98 x 10^−4^–3.38 x 10^−3^	4.15 x 10^−3^	Rvir	3.69 x 10^−3^	2.35 x 10^−3^–5.02 x 10^−3^	3.99 x 10^−3^
Rcl3	8.28 x 10^−3^	2.32 x 10^−3^–9.52 x 10^−3^	6.41 x 10^−3^	Rsag	4.55 x 10^−3^	1.09 x 10^−3^–6.66 x 10^−3^	2.48 x 10^−3^
Rcl5	4.26 x 10^−3^	2.61 x 10^−3^–6.44 x 10^−3^	4.35 x 10^−3^	Rxil	5.14 x 10^−3^	2.18 x 10^−3^–8.09 x 10^−3^	2.94 x 10^−3^

Parameters N, T, M, and R denote population sizes, times, migration rates, and growth rates. Population sizes are given in number of individuals and times in number of generations. The admix parameter gives the proportion of admixture. Given are the maximum likelihood parameter point estimates and in the case of the “Admixture prior to sympatric speciation model” also the 95% confidence intervals obtained from nonparametric bootstrapping. Species names are abbreviated by their first three letters.

^a^Migration among sympatric species is assumed to be identical across all directions. Migration rates denote the probability of an allele to coalesce in another deme per generation. Note that migration rates cannot be readily converted to number of individuals in growing populations.

^b^Migration from the source population into the crater lake species in forward time (identical across species).

Although the secondary contact model had a lower likelihood we report the parameter estimates for comparability (Table 3). For many parameters such as the timing of the bottleneck and population sizes we obtained similar estimates as for the model above, although some of the current sizes deviate. Most importantly, according to this model the first colonization would have happened ca. 1,650 and the second colonization ca. 1,150 generations ago. Around 910 generations ago the population stemming from the secondary colonization would have received about 90% of its gene pool from the primary founder population, replacing it almost completely. The three benthic species would then have diverged approximately 740 generations ago.

#### Crater Lake Xiloá

In the most supported model (admixture prior to sympatric speciation) of L. Xiloá (Table 3) the bottleneck in the source population of L. Managua happened ca. 1,420 generations ago, during which the population decreased from about 18,300 to only approximately 1,850 individuals, recovering to about 287,300 individuals at present. The colonization of L. Xiloá from L. Managua took place ca. 1,320 generations ago by a small founder population of about only 150 individuals. Interestingly, the admixture event happened at around the same time as in L. Apoyo, about 890 generations ago, but the admixture proportion was much higher with ca. 29%. Again similarly to L. Apoyo, the first speciation event happened only a few generations after the admixture event. In this case it led to the species *A*. *amarillo*, *A*. *viridis*, and the ancestral lineage of the other two species, which diverged in turn ca. 750 generations ago. The current population sizes of *A*. *amarillo* and *A*. *viridis* are similar (ca. 34,500 and 35,500 individuals) and larger than the sizes of *A*. *sagittae* and *A*. *xiloaensis*, which are in turn similar (14,800 and 12,800). Migration rates among the sympatric species are 8.8 x 10^−5^ and from L. Managua into L. Xiloá 1.7 x 10^−5^.

According to the hypothetical model of secondary contact the two colonizations would have happened 1,140 and 870 generations ago, with an admixture event of 73% ca. 850 generations ago and a simultaneous divergence of the two lineages into the four species 825 generations ago. Estimates of population sizes and migration rates were similar to those of the sympatric speciation model (Table 3).

## Discussion

Whether geographical isolation is required for (the initiation of) speciation continues to be one of the most controversially discussed topics in evolutionary biology. Sympatric speciation is the most extreme case of primary divergence-with-gene-flow, in which geographic barriers play no role in reducing gene flow [17, 18]. While theoretically possible, only few putative empirical examples exist and, together with Palm trees and some other plant lineages on Lord Howe island [39, 70], crater lake cichlids in Cameroon and Nicaragua have been among the most widely-accepted examples [18, 22]. Moreover, a recent study provided evidence that the divergence of two eco-morphs of cichlids in a crater lake in Tanzania happened in sympatry [71]. Yet, evidence for complex phases of secondary contact and gene flow among crater lake radiations and riverine populations of Cameroonian cichlids was provided recently [34] and some criticism of sympatric speciation in Nicaraguan Midas cichlids was expressed initially: the criticism was mainly concerned with the fact that not all species of Midas cichlids in the crater lake and source lake were taken into account and that no explicit explanation for the intermediate position of a species in multivariate genetic space was given [27]. In this study we took previous and more recent concerns [34, 53] into account and evaluated the evidence for putative periods of allopatry in two radiations of Midas cichlids using a comprehensive genomic data set. Since Midas cichlids provide the rare advantage that the actual source populations of the crater lakes are known, we were able to reconstruct the demographic history of two crater lake radiations. This allowed us not only to test for differential admixture of crater lake species—for which we found no evidence—but to detect a secondary colonization (admixture) from the same respective source population prior to the onset of the radiations. This admixture event would have been otherwise difficult to detect as it results in equal proportions of shared ancestry among all species within a radiation.

### Species and genetic clusters

Any argument for or against sympatric speciation has to rest on a valid taxonomic assignment [27] and while we agree that it is important to take all species in a respective radiation into account the current taxonomy in L. Apoyo has been in conflict with genetic data. For example, only a single species formed a monophyletic group in [53] and [49] found the highest support for only two genetic clusters. Thus we decided to use a more objective approach and assign individuals to genetic clusters—essentially applying a genetic cluster species concept [57]–and adhere to these clusters for all subsequent analyses. But we note that the assignment based on morphology fits the genetic signature in all cases of *A*. *zaliosus* in L. Apoyo and almost all cases in L. Xiloá. And even in the case of the genetic clusters in L. Apoyo there are clear trends. For example, all individuals of *A*. *astorquii* are assigned to cluster 2, and eight of the nine individuals in cluster 4 are *A*. *globosus*. Consequently, our results do not imply that there are no morphologically distinct species of Midas cichlids, but rather that the assignment based on morphological criteria alone is often difficult in these young radiations with ongoing hybridization. Nonetheless, our data do not support the current six-species taxonomy in L. Apoyo since we only find strong support for five instead of six genetic clusters. It is worth mentioning though that our results only apply to our data set and that it cannot be ruled out that more genetic clusters exist. In L. Xiloá our results agree with previous studies that have reported four genetic clusters corresponding to the currently described four species [48, 49]. Interestingly, a high number of admixed individuals between *A*. *sagittae* and *A*. *xiloaensis* is already apparent in these studies, although this was not the focus of their discussion.

### Hybridization among sympatric species

The occurrence of some gene flow between the sympatric species is not unexpected, as reproductive barriers are thought to be incomplete and mainly based on mate choice (pre-mating) and divergent selection against hybrids (extrinsic postzygotic). No intrinsic incompatibilities are known to occur in Midas cichlids [72] and species can be easily crossed in the laboratory [73, 74]. That we find so much hybridization between the limnetic *A*. *sagittae* and the benthic *A*. *xiloaensis* is puzzling. If these two species arose by ecological speciation one would expect hybrids to have a reduced fitness and be thus less frequent [75–78]. We can think of two main possible explanations for the existence of the hybrid group: the two species *A*. *sagittae* and *A*. *xiloaensis*, which seem to have diverged only ca. 750 generations ago, might still be in an early stage of the speciation continuum [23] and reproductive barriers are weaker than between other species. Alternatively, the hybrid group itself could be in the process of becoming a stable and distinct population. In other words we could be witnessing the early stages of another speciation process due to ecological niche partitioning. Our genetic data provide conflicting evidence for the two alternative scenarios. The hybrid group did not form a distinct cluster in our *Admixture* analysis, yet it formed the sister group of *A*. *xiloaensis* in our phylogenetic analysis and did not exhibit an intermediate position between the two species. We acknowledge, however, that the latter result was probably affected by the admixture proportions of the randomly selected individuals used to infer the phylogeny: a *post hoc* examination showed that the average admixture proportions of the four used hybrid individuals were slightly in favor of *A*. *xiloaensis* (51.7% of their ancestry) over *A*. *sagittae* (48.3% ancestry). This could explain why the hybrid group was resolved as the sister group of *A*. *xiloaensis* and did not result in a trifurcation. On the other hand, overall genetic differentiation between the two species *A*. *sagittae* and *A*. *xiloaensis* was relatively high (S2 Table), which might not be expected if there was a lot of ongoing gene flow between them. More detailed genomic analyses as well as ecological experiments beyond the scope of this study are necessary to determine the fitness and ecological niche of hybrids compared to both parental species.

### Species in the two radiations are equally related to both great lake species

The two large and old source lakes contain two species of Midas cichlids and sympatric speciation requires that none of the species in the respective crater lakes are more closely related to either of the species in the source lakes than the other sympatric species are [27]. Our phylogenetic analyses suggest that the species in the great lakes are sister species and thus equally distantly related to each of the species in the crater lake radiations. They are also equally distant in genetic space in our global PCA (Fig 1). This pattern either suggests that the two species in the great lakes only diverged after the crater lakes had been colonized from their shared ancestral population, or that we do not have enough power to resolve the exact relationships with our current data set; the two species in the great lakes are genetically almost not distinguishable (S2 Table) [52]. Only the latter case would make an interpretation in regard to sympatric speciation more difficult. If the crater lakes had been colonized by a set of two species, two (but not all) of the species in the respective radiations could theoretically simply be the descendants of the two founding species. Efforts to characterize the genomic differences between the two great lake species are currently underway and diagnostic haplotypes might help to finally resolve whether these crater lakes were colonized by either one or both of the species. Yet, the fact that no fish resembling *A*. *labiatus* with its characteristic hypertrophied lips exists in these crater lakes (they have only anecdotally been reported to occur in L. Xiloá) speaks in favor of a colonization by *A*. *citrinellus* alone. We note that fish resembling *A*. *labiatus* do occur at considerable frequencies in two other crater lakes, L. Masaya and L. Apoyeque [79]. Hence, the ecological niche (foraging in rocky crevices) that *A*. *labiatus* is adapted to [46] is probably present in crater lakes Apoyo and Xiloá as well and if *A*. *labiatus* colonized these crater lakes it is difficult to conceive of why their phenotype would have changed completely. We further note that *A*. *labiatus* occurs much less frequently in the great lakes than *A*. *citrinellus* (ca. 5%) and it is therefore not unlikely that *A*. *labiatus* never colonized these crater lakes while *A*. *citrinellus* did.

In conclusion, while we cannot rule out at the moment that two of the sympatric species in the crater lake radiations are the result of a double-colonization by the two species from the great lakes (if they diverged before the colonization of the crater lakes), we think the fact that no clear traces of an *A*. *labiatus*-like phenotype are present in these two crater lakes makes a colonization by only one species more parsimonious.

### Multispecies outcomes of sympatric speciation

Besides establishing the relationship between the two species in the great lakes and the crater lake species we were further interested in the branching pattern within the radiations. In L. Apoyo the cloudogram resembles a starlike phylogeny with an almost simultaneous split of all five species (Fig 3A). Yet, *A*. *zaliosus* is the first species to branch off the stem lineage, albeit only slightly before the other species. This split is also supported in our demographic models and is consistent with previous studies [49, 52]. However, neither our phylogenetic tree, network, nor demographic models can unambiguously resolve the relationships among the other four endemic species from L. Apoyo. Similarly, the tree in L. Xiloá remains only partially resolved with a simultaneous split of *A*. *amarillo*, *A*. *viridis*, and the ancestor of *A*. *sagittae* and *A*. *xiloaensis* (Fig 3B). The sister relationship of the latter two is again consistent with our earlier work [49], supporting the interesting conclusion that the limnetic-benthic divergence happened via non-parallel routes in the two parallel adaptive radiations of the crater lakes. Yet, in our previous phylogenetic analysis [49] *A*. *amarillo* splits off first in all bootstrap replicates. The discrepancy with these current results could be due to the fact that the former phylogeny was based on a concatenated SNP matrix, which may be problematic in this young species complex, where shared ancestral variation and incomplete lineage sorting prevail [80].

Our analyses in this study explicitly take incomplete lineage sorting and in the case of the demographic models also gene flow and changing population sizes into account. Thus, we are led to support the hypothesis that some of the speciation events happened simultaneously and represent hard polytomies, as has been recently suggested to occur in birds [81]. And even if the splits did not happen strictly simultaneously they seem to have occurred in extremely rapid succession, which suggests that ecological interactions among the incipient species may have played a role. The possibility of a multispecies outcome of sympatric speciation was proposed based on a theoretical model ten years ago [82], strikingly invoking crater lake cichlids as an example where it might have occurred. Indeed, our phylogeny of L. Apoyo resembles the outcome of a simulation, in which one panmictic population diverges into six species after only ca. 400 generations [82]. Thus, we propose that Midas cichlids represent, to our knowledge, the first empirical case of a multispecies outcome of sympatric speciation. The model was, however, one of ‘pure’ sympatric speciation and if the admixture event prior to radiation that we detected is real and did facilitate speciation in sympatry the theoretical model may not directly correspond to the situation in Midas cichlids.

### Evidence for primary divergence-with-gene-flow versus secondary contact

Unequal levels of shared ancestry with an outgroup or the source population can be indicative of a past period of allopatry of sympatric species followed by introgression upon secondary contact. This pattern would be expected to be reflected in intermediate positions of certain species along the major axes of genetic variation in a PCA [27, 34, 83]. In our global PCA (Fig 1) all individuals from L. Apoyo are equidistant to the source population, consistent with sympatric speciation. In L. Xiloá, however, two species are closer to the source population than the other two. While this pattern might suggest secondary contact and hybridization, the *f3-statistics* do not support this explanation. This signal of admixed ancestry did also disappear in the *Admixture* analyses when assuming more than four clusters (S1 Fig). Instead, we propose that this pattern in the global PCA might rather reflect a difference in population sizes; our demographic models suggest that the population sizes of *A*. *amarillo* and *A*. *viridis* have been larger than the other two species and they may have thus retained more of the ancestral variation. Thus, altogether our phylogenetic and genetic clustering results are consistent with sympatric speciation and provide no evidence for an initial divergence of the sympatric crater lake species in geographic isolation followed by introgressive hybridization (a scenario of secondary contact).

Yet, *f3-statistics* may not have enough power to detect admixture in species that have subsequently experienced a considerable amount of genetic drift [60, 61, S1 Text therein], which might be the case in Midas cichlids. Furthermore, neither the clustering or phylogenetic methods, nor *f3-statistics* will detect multiple colonizations from the same source population (admixture) that happened prior to the onset of the radiation, as all species within the radiation would share the same amount of shared ancestry and drift paths compared to the source population. Thus, we formulated the most plausible hypotheses for the different evolutionary scenarios in demographic models and evaluated their evidence using information-theory-based criteria [69]. The main three models we aimed to compare were: sympatric speciation after a single colonization, sympatric speciation after a secondary colonization (admixture prior to sympatric speciation), and two waves of colonization followed by admixture (secondary contact and introgressive hybridization). The first two models are scenarios of primary divergence-with-gene-flow, whereas the latter one models secondary gene flow after an initial period of allopatry.

For both radiations the respective models of sympatric speciation after a single colonization had essentially no support and an admixture event from the source population into the crater lakes prior to sympatric speciation is strongly supported. Yet, consistent with our clustering and phylogenetic analyses as well as *f3-statistics*, the evolutionary history of both radiations is better modelled by a scenario of primary divergence-with-gene-flow than by an initial period of allopatry of the crater lake species themselves: in L. Apoyo sympatric speciation after an admixture event is 2.5 times more likely than a scenario of secondary contact. Furthermore, even though the likelihood for secondary contact is not negligible the scenario seems biologically less plausible. According to the parameter estimates the population of secondary colonizers (i.e. the ancestral population of the four benthic clusters) would have received 90% of its gene pool from the established population (i.e. the lineage of *A*. *zaliosus*), but only about 250 generations after they arrived in the crater lake. For the radiation in L. Xiloá the evidence is clearly in favor of sympatric speciation after admixture, which is 70 times more likely than secondary contact. The fact that the likelihood ratio between the model of sympatric speciation and secondary contact is so much higher in L. Xiloá than in L. Apoyo could be due to the fact that *A*. *zaliosus* in L. Apoyo is much more distinct from the other sympatric species than any of the species in L. Xiloá are compared to each other. Furthermore, we had slightly more data in the site frequency spectrum (SFS) of L. Xiloá and thus potentially more power to distinguish between the models.

### Possible impact of admixture from the source populations in facilitating speciation in sympatry

Overall, for both radiations our data provide more support for a model of primary divergence in sympatry than one in which already partly diverged populations diverged further and speciated upon secondary contact. However, an admixture event from the source population into the stem lineage of the crater lake flocks (before the species diverged) is strongly supported in both cases compared to the respective models of sympatric speciation after a single colonization, thus opening the possibility of sympatric speciation after formation of a hybrid swarm [34, 36]. The fact that the admixture event happened in both radiations shortly before the first speciation event makes it tempting to assume a causal relationship. We think that this is certainly possible, but we warrant caution at this point. It is an interesting hypothesis that the 4% admixture into L. Apoyo from the same ancestral lineage after only about 800 generations of separation provided the genetic substrate to initiate sympatric speciation. But only when the traits involved in reproductive isolation and their genetic basis is identified can a causal relationship be investigated. Furthermore, distinguishing between the causes of shared polymorphisms remains inherently difficult [84–87]. Once a trinucleotide substitution matrix [88, 89] is available for cichlids, future studies making use of information about ancestral and derived allelic states, that is, using the more powerful derived SFS, should be used to evaluate our results. Moreover, whole-genome data will likely increase the power to test the different hypotheses due to a higher number of segregating sites and information about the size of linkage blocks [90]. The 29% admixture into L. Xiloá seem more likely, at least probabilistically, to have had an impact. In any case, the admixture events would primarily explain the first speciation events in the two radiations and further speciation might have been sympatric in the ‘pure’ sense. Yet, we acknowledge that further speciation events could also have been driven by bouts of ecological interactions and complex sorting of partial reproductive incompatibilities, once two or more (incipient) species had evolved [36].

### Bottlenecks in the source populations coincide with tectonic events

The support for a bottleneck in the great lake populations around 1,500 and 2,000 generations ago was unexpected, yet it is not inconceivable that major geological events in this tectonically active area of Nicaragua have strongly affected the fauna in the lakes. Indeed, there is geological evidence for an underwater eruption of a volcano in L. Managua that caused a tsunami only about 3,000–6,000 years ago [91] and other possible tsunamis in L. Nicaragua triggered by debris avalanches [92]. Assuming a generation time of two years we propose that the inferred bottleneck coincides with such an event. The signal of exponential growth in the great lakes after such an event is not unexpected and also the inferred exponential growth of the crater lake populations after colonization by a small founder population seems biologically sensible. Nonetheless, a signal of population growth can be falsely inferred for several reasons. Technical reasons, such as sequencing or PCR-based errors leading to an excess of singletons seem unlikely, as growth was also supported with a more stringent threshold of 15x read depth and since we used a low number of amplification cycles, performed ten PCR replicates, and used a high-fidelity polymerase for genomic library preparation. Multiple-merger coalescent events [93] and background selection [94], however, cannot be ruled out to have affected the analyses. Yet, the site frequency spectrum (SFS) contains often enough information to distinguish between multiple-mergers and exponential growth [95] and methods to jointly infer the demographic history and the effects of selection are an active and promising area of research that may help to sort out the relative effects of selection and demography [94, 96].

### Recent colonization and extremely fast speciation

The inferred colonization and divergence times for these endemic crater lake cichlid species are much lower than we anticipated. Considering that L. Apoyo is ca. 24,000 and L. Xiloá ca. 6,100 years old [97], our results would imply that especially L. Apoyo has been devoid of a stable population of Midas cichlids for much of its history. Previous studies have reported divergence times that are closer to the age of the lakes, yet these studies were based solely on a single mtDNA marker for which calibration times and molecular clock rates are uncertain or debated [50, 54]. Moreover, using a local substitution rate that was calibrated by equating the geological age of L. Apoyo with a signal of population expansion (mismatch distribution of mtDNA) as a proxy for divergence time [98] might have been a too strong assumption. Uncertainty about the substitution rate might also be a source of error in this study. Similarly, the ratio of monomorphic to polymorphic sites is important for obtaining absolute estimates and is to some extent affected by the way the data has been processed. Too strict filtering can lead to an underestimate of the number of polymorphisms and bias the absolute estimates. However, while our absolute estimates may change depending on the substitution rate and data filtering criteria, the relative values and the model selection procedure should not be affected by this. Assuming that the substitution rate is approximately correct and the effect of data filtering unbiased and negligible, with ca. 1,690 and 1,320 generations for L. Apoyo and L. Xiloá, the ages of these two radiations are much younger than previously thought. Alternatively, we cannot rule out the possibility that older populations of cichlids in these crater lakes might have been almost or completely exterminated—for example by volcanic activity (although there is no geological evidence for this)—and these earlier populations or species were replaced only recently by the extant radiations that are less than 2,000 generations old. Recurrent mass extinctions due to volcanic activity have possibly occurred in other crater lakes such as Lake Apoyeque [79] and such events would lead to a loss of information stored in the SFS [68] and could thus bias our estimates downwards. The same argument applies to the bottleneck in the source populations, yet our simulations suggest that we can correctly infer divergence times that happened before the bottleneck. Ultimately, fossils from the beds of the crater lakes might further inform on this issue.

Cichlid fishes in general exhibit one of the fastest known speciation rates [99] and, acknowledging the caveats described above, our data suggest that speciation rates in Midas cichlids might even be the fastest reported yet. According to our estimates it took only around 1,000 and 600 generations from the time of colonization to the last splits leading to the five species in L. Apoyo and four species in L. Xiloá, respectively. Such a rate of speciation is unprecedented, even though it might be considered that speciation is not fully completed yet as there is still some level of ongoing gene flow—at least between a subset of these species. Ecological speciation in general can commence very rapidly [100] and a theoretical model of Midas cichlids showed that sympatric speciation can happen in less than 20,000 generations [101]. Moreover, in the model of [82] five species evolved in as little as ca. 400 generations–quite similar in both number and timing as we have inferred in the case of these two crater lake adaptive radiations. Both of these models are based on several assumptions and investigating whether these are met in Midas cichlids will require future behavioral and ecological research.

### Conclusion

In this study we reconstructed the demographic history of two endemic radiations of Midas cichlids inhabiting the small and isolated crater lakes Apoyo and Xiloá to infer whether complex periods of geographic isolation may have facilitated speciation within the two radiations. Apart from this main objective our large genome-wide data set suggests that most of the species evolved in a burst of speciation, thus making these two radiations of cichlids, to our knowledge, the first empirical examples of multispecies outcomes of sympatric speciation [82]. Moreover, these radiations of nine species took place within only about a thousand generations, making them some of the fastest speciation rates reported so far. Unlike recent evidence presented for Cameroonian crater lake cichlids [34], our population clustering and phylogenetic analyses are consistent with a scenario of primary divergence-with-gene-flow (sympatric speciation) of the crater lake species. Interestingly though, our models do provide evidence for a secondary colonization from the source population that happened shortly before the species radiated in both crater lakes. Whether Midas cichlids represent therefore a good case of sympatric speciation may ultimately depend on the definition of sympatric speciation [19–21]. The species flocks of Midas cichlids in the Nicaraguan crater lakes arose via sympatric speciation in the sense that their divergence happened most likely in a geographic setting that does not offer geographic barriers to gene flow. Yet, if the admixture event from the source population was instrumental for seeding speciation in sympatry by providing some of the genetic variation involved in reproductive isolation, then a short period of geographic isolation would have been involved in speciation. This would make the radiations of Midas cichlids no longer a case of ‘pure’ sympatric speciation, similarly to the sympatric divergence of apple maggot flies in North America for example [35]. Whether the admixture event was essential for speciation remains to be elucidated. If confirmed, this could partly explain how Midas cichlids have speciated so rapidly in sympatry. It would also exemplify that the term ‘primary divergence’ may have a different meaning when applied at the level of populations and incipient species or at the level of individual genetic variants that distinguish them while the rest of the genome can be exchanged freely [102]. Overall, rather than adding to the debate over whether speciation conforms to a single category of speciation or not we think the results presented here open up a new and exciting hypothesis of how speciation may have happened in the extremely young and repeated radiations of Midas cichlids.

## Materials and Methods

### Ethics statement

Sampling was approved and conducted in accordance with the regulations of the local authorities, the Ministerio de Ambiente y Recursos Naturales, Nicaragua (MARENA).

### Sampling and ddRAD sequencing

Fish were collected in the field in 2001, 2003, 2005, 2007, 2010 and 2012 with gill nets or by harpooning. Specimens were photographed in a standardized way and tissue samples from fin or muscle were taken and preserved in pure ethanol. Genomic data were generated using double-digest RAD sequencing [103] following an in-house protocol [104] with minor modifications. Briefly, for each individual 600 ng DNA template were digested with the restriction enzymes PstI-HF (NEB) and MspI (NEB). The success of every single digestion reaction was visually inspected on a 2% agarose gel and samples showing a heterogeneous fragment distribution (e.g. due to incomplete digestion or degradation) were replaced. After ligation of individually-barcoded adaptors [provided in ref. 104] individuals were combined into pools of 50 samples. Fragments in a range of 320–500 bp were selected using Pippin Prep technology (Sage Science, Beverly, MA) and amplified in replicates of ten PCRs per pool, running for ten cycles, using a Phusion high-fidelity polymerase (NEB). Oligonucleotide dimers were removed by gel electrophoresis and fragment size distribution was inspected with an Agilent 2100 Bioanalyzer machine. Finally, genomic libraries (pools) were single-end sequenced for 101 cycles using Illumina HiSeq 2000 technology at the genomics core facility of TUFTS University (Boston, MA).

### Mapping and genotyping

Sequence quality was inspected with *FastQC* and no systematic bias or quality drop-off at the end of the reads was observed. Thus, no trimming was performed. Individually-barcoded reads were de-multiplexed using the *process_radtags* script included in the *Stacks v*.*1*.*29* software pipeline [105, 106]. Reads containing uncalled bases and/or showing an average quality score of less than 25 in a sliding window of 10% total read length were discarded. The remaining reads were mapped to an anchored in-house genome assembly of an individual of *A*. *citrinellus* from Lake Nicaragua [49] with *bwa v*.*0*.*7*.*12* [107]. Reads mapping to several positions in the genome, containing soft-clipped positions, or showing a mapping quality of less than 25 were discarded using custom bash scripts. Genotyping was conducted with *Stacks* using a minimum of five reads to form a locus. Based on population level information, the *rxstacks* correction module of *Stacks* was used to remove loci being confounded in more than 25% of individuals, or showing an excess of haplotypes within populations. This module furthermore corrects individual genotype calls based on population information. Genotypes were called setting an upper bound of 0.05 for the error rate and using a 5% significance level cut-off (non-significant likelihood ratios of genotype models resulted in uncalled genotypes). At each locus and individual, log-likelihood values for each genotype call (every nucleotide position) were summed up and individual genotype calls at loci with an overall log-likelihood of less than -10 were filtered out and did not contribute to any subsequent analyses. If an individual is, for example, unambiguously homozygous across the whole length of a locus the respective log-likelihood value will be zero. Similarly, if a heterozygous position is supported by an equal representation of alleles, the log-likelihood for the call will be close to zero. Loci with many poorly supported genotype calls (e.g. due to sequencing errors) will exhibit more negative log-likelihood values. The cut-off value of -10 was chosen based on the empirical distribution of log-likelihoods in our data set. On average 70,538 ± 17,191 (sd) loci were obtained per individual with a mean coverage of 13.8 ± 4.9 (sd) reads per locus and individual. Individual- and population-level information on number of loci, average coverage per locus, proportion of missing data included in the matrix used in the global PCA (Fig 1) and Admixture analysis (S1 Fig), as well as genomic library IDs are provided in S7 Table. Our data exhibited an excess number of polymorphisms in the last two positions of the reads and loci with polymorphisms in these positions were thus excluded (i.e. blacklisted) from all subsequent analyses. In an attempt to account for hidden paralogy, loci deviating from Hardy-Weinberg-Equilibrium (HWE) (5% significance level) or containing more than three SNPs within a population were excluded from further analyses. HWE exact tests [108] were performed in *Plink v*.*1*.*19beta* [109]. Hybrid individuals were treated together as a separate group and no HWE tests were conducted in this group. Furthermore, unless noted otherwise, only loci that were genotyped in at least six individuals per population were used in subsequent analyses.

### Population structure

Population structure in our data set was explored with the model-based approach of *Admixture v*.*1*.*23* [56] and with model-free principal component analyses (PCA) as implemented in the *Eigensoft v*.*5*.*0*.*2* package [55]. Both methods were applied in a hierarchical design. First, all samples were included in one analysis (‘global’). In a second step, samples from the two crater lakes were analyzed separately to investigate population structure within lakes (‘intralacustrine’) in more detail. *Admixture* was run from 1–18 predefined clusters (K) in the global analysis. In the intralacustrine analyses *Admixture* was run for 1–8 clusters. Statistical support for the different number of clusters was evaluated based on ten rounds of the implemented cross validation technique. Missing data in the PCAs were accounted for by solving least squares equations (applying the *lsqproject* function). Statistical significance of principal components was determined by means of the implemented Tracy-Widom statistics. PCAs were visualized in *R v*.*3*.*1*.*2* [110] using the *scatterplot3d* library [111]. For both approaches only one SNP per locus was used to reduce the effect of non-independence (linkage) among markers. Overall pairwise genetic differentiation was calculated in *Arlequin v*.*3*.*5*.*1*.*3* [112, 113] and statistical significance was assessed by means of 10,000 permutations. The same data set that was used for the global PCA and *Admixture* analyses went into this analysis.

### Morphological analyses

We examined body shape differentiation among populations of the two crater lakes using the body height index (BHI) and geometric morphometrics. Data for both measures were obtained from standardized pictures. The BHI is defined as the ratio of body height divided by standard length and is a simple measure to capture the main morphological differentiation between the elongated limnetic and high-bodied benthic species. For geometric morphometrics seven homologous body landmarks were digitized in *TPSDIG 2*.*17* [114] for all individuals of *A*. *sagittae*, *A*. *xiloaensis*, and the hybrid group in L. Xiloá. Landmarks are a subset (labels 1, 6, 9, 10, 12, 14, 15) of previously defined positions [44, Fig 2] that capture the main differentiation in body shape between the focal species. Shape analyses were performed in *MorphoJ 1*.*03d* [115]. Landmarks were first aligned using a full Procrustes superimposition, which involves scaling all shapes to unit centroid size, translation to a common position, and rotation to minimize the Procrustes distance between landmark configurations [116, 117]. Allometry is common in fish and thus morphology and total body size are typically related [117]. Therefore, a multivariate regression of body shape (Procrustes coordinates) on size (centroid size) was used to correct for allometric effects. Regression residuals were then used for all downstream geometric morphometric analyses. Individual variation in body shape across and within species was visualized using a PCA on the regression residuals.

### Tests for differential admixture and *Treemix* trees

First, a maximum likelihood phylogenetic tree was built from allele frequency data using *Treemix v*.*1*.*12* [61]. Support for the tree topology was assessed by means of 1,000 bootstrap replicates using a block size of 20 adjacent SNPs. Trees were rooted with *A*. *citrinellus* from Lake Nicaragua. Up to four migration events were fitted on the tree. Admixture between populations was formally tested with *f3*-*statistics* [59] implemented in the *threepop* software of *Treemix*. Standard errors were calculated in blocks of 20 adjacent SNPs. Only SNPs assigned to the 24 linkage groups of our reference genome were used in *Treemix* and *threepop* analyses.

### Phylogenetic trees and networks

Phylogenetic trees were built using the Bayesian method implemented in *SNAPP v*.*1*.*10* [62]; an add-on package of *BEAST v*.*2*.*2*.*1* [118]. Due to the computational demand of *SNAPP* only four randomly selected individuals per species were used and trees for the two crater lakes and their respective source populations were built separately. Backward and forward mutation rates (u+v) were estimated from the stationary allele frequencies in the data (u = 0.6196; v = 2.5907 for L. Apoyo and u = 0.6596; v = 2.0661 for L. Xiloá). Only one SNP per locus and only SNPs genotyped in all individuals were used. Each analysis was run for more than five million generations, discarding the first 10% as burn-in. Trace files were inspected with Tracer v.1.6 [119] and effective sample sizes were higher than 200 for all parameters. Trees were visualized with *DensiTree* [120].

In a second approach we built individual-based phylogenetic networks with *SplitsTree v*.*4*.*13*.*1* [121]. Similar to the phylogenetic trees, the networks were built separately for the two crater lake radiations and their respective sources. Individual genotype calls were transformed from VCF to Nexus format using custom scripts and networks were built using the NeighborNet method based on uncorrected P distances.

### Demographic inference based on coalescent simulations and the SFS

The demographic history was inferred using the information contained in the multidimensional site frequency spectrum (SFS) and *fastsimcoal v*.*2*.*5*.*2*.*3* [66]. Briefly, *fastsimcoal2* performs coalescent simulations under an arbitrarily complex predefined demographic model and then uses a conditional maximization (ECM) algorithm to optimize each parameter in turn to maximize the likelihood given the data. Demographic models are not restricted to a certain number of populations and can include a variety of demographic events such as migration, population size changes, population splits and admixture events. In an attempt to reduce the effect of selection, loci presumably located in coding regions were excluded; these loci were identified via a *blastn* search against a compilation of transcriptomic data from various species and tissues of Midas cichlids [45, 122, 123]. Furthermore, only one SNP per locus was used to reduce the effect of non-independence of markers [124]. The SFS was created in the following way: data were parsed from variant call format (VCF) files using a custom python script and transformed into the MSFS using a modified script available from *δaδi* [67]. Since no trinucleotide substitution matrix is available for cichlids to correct for ancestral misidentification [88, 89] we used the minor (folded) site frequency spectrum. Initially, simple one-population models were run for both source lake populations. Subsequently, each crater lake species was analyzed together with its respective source population in two-population models. Finally, for both crater lakes each four species were analyzed jointly with the source population in five-population models. Including all five sympatric species in one analysis in the case of Apoyo was not possible as *fastsimcoal2* is currently limited to handling SFS files of up to one million entries and including another population would have meant reducing the sample size to only four samples per population. The number of entries in the multidimensional SFS is the product of the number of alleles plus one (for the state of zero) per population. In the case of L. Apoyo we excluded cluster 4 as we only had nine individuals in our data set. In L. Xiloá all four sympatric species could be included in one analysis; the hybrid group was not considered in these analyses. To alleviate the problem of missing data in building the SFS from RADseq data, sample sizes were projected downwards to a certain size using *δaδi*’s projection function [67]. In one- and two-population models the source populations were projected down to 50 alleles (25 individuals), except for *A*. *labiatus* from L. Managua (projected to 30 alleles), and the crater lake species each to 30 alleles, except for clusters 3, 4, and 5 in Apoyo, which were projected down to 20, 14, and 20 alleles, due to their small sample sizes, respectively. In the five-population models sample sizes had to be projected down to 18 alleles for the source populations and 14 alleles for each of the crater lake species due to the limitation of a maximum of one million entries in the SFS. Note that the projected samples sizes were used as a minimum threshold to create the VCF files, that is, a locus for which fewer samples were genotyped than the targeted sample size for projection in any one population was excluded. This filter was applied to both polymorphic and monomorphic loci, which is crucial to obtain the correct ratio of monomorphic to polymorphic sites, by which all demographic parameters are scaled. In more detail, the number of monomorphic sites was added manually to the SFS and theoretically equals the respective number of loci times the 89 potentially variable sites (obtained by subtracting the 5 bp of restriction site and the 2 blacklisted sites from the 96 bp reads) minus the number of segregating sites. Yet, using only one SNP per locus decreases the ratio of polymorphisms and thus biases the estimates. This bias was corrected for by first calculating the ratio of monomorphic to polymorphic sites using all SNPs. The resulting number of monomorphic sites is then the number of SNPs (one per locus) multiplied by this ratio. To convert the inferred parameters into demographic units, a substitution rate of 7.5 x 10^−9^ per site and generation similar to a recent estimate from nine- and three-spine sticklebacks was assumed [125]. For each demographic model at least 25 independent *fastsimcoal2* runs with relatively broad prior search ranges for the parameters were conducted. If several competing models returned similar likelihoods or we were interested in the maximum likelihood parameter estimates the number of runs was increased. Likelihoods are approximated and increasing the number of runs thus increases the chance of minimizing the error [66]. In this case the prior search range was adjusted to better accommodate more likely values of the parameters. Especially in the case of more complex models and time parameters reducing the prior search ranges often enhanced convergence. But note that prior bounds only define initial search ranges and are not to be understood like priors in a Bayesian approach. Only the lower bound is an absolute boundary in *fastsimcoal2*. The upper bound can increase each round. Each run consisted of 20–50 rounds of parameter estimation via the ECM algorithm with a length of 100,000–250,000 coalescent simulations each (increasing by 5,000 steps each round). The relative fit of the different demographic models to the data was evaluated by means of the Akaike Information Criterion (AIC) after transforming the log_10_-likelihood values to ln-likelihoods. Following [66] 95% confidence intervals were calculated by parametric bootstrapping. Bootstrap replicates (n = 25) were obtained by simulating minor site frequency spectra using the same overall corrected sequence length as the empirical data (unlinked regions of 89 bp) and according to the maximum likelihood parameter point estimates followed by re-estimating the parameters.

## Supporting Information

S1 Fig Global admixture analyses. A) Shown are the result assuming the same number of clusters as lakes (K = 4) and the most supported number of clusters (K = 12). Note that K = 9 is equally well supported, but results for K = 12 are shown as this more closely reflects the number of populations in our data set. B) Support for all runs assuming *a priori* 1–18 clusters. A lower cross-validation error indicates higher support.
(TIF)

S2 Fig Cross-validation errors for intralacustrine admixture runs. For both crater lake radiations between 1–8 different clusters were assumed *a priori*. A lower cross-validation error indicates higher support.
(TIF)

S3 Fig Geometric morphometrics. Shown are the first two axes of a principal component analysis based on seven homologous landmarks. Individuals genetically assigned to the hybrid group in L. Xiloá occupy an intermediate position between the limnetic species *A*. *sagittae* and the benthic species *A*. *xiloaensis*.
(TIF)

S4 Fig Body height index (BHI). A) In L. Xiloá the limnetic species *A*. *sagittae* exhibits the most elongated body shape (lower BHI). The hybrid group is intermediate to *A*. *sagittae* and the rest of the benthic species. In L. Apoyo the limnetic *A*. *zaliosus* is markedly more elongated than the other four benthic species. B) Schematic illustration of the BHI.
(TIF)

S5 Fig Genomic differentiation among sympatric species in L. Apoyo. For all ten pairwise comparisons the overall distribution of F_ST_-values (density plot) and the genetic differentiation across the 24 linkage groups of the reference genome are given. Markers with a minor allele frequency (MAF) below 5% were excluded. The number of markers for each comparison is given in S3 Table.
(TIF)

S6 Fig Genomic differentiation among sympatric species in L. Xiloá. For all six pairwise comparisons the overall distribution of F_ST_-values (density plot) and the genetic differentiation across the 24 linkage groups of the reference genome are given. Markers with a minor allele frequency (MAF) below 5% were excluded. The number of markers for each comparison is given in S3 Table.
(TIF)

S7 Fig Treemix graphs. Maximum likelihood trees based on allele frequencies at 13,477 loci located on the 24 linkage groups with A) no migration (m = 0) or C) E) G) I) fitting up to four migration events (m = 1–4) on the tree. Lakes are indicated by color-shading. Nodes that have 100% bootstrap support are indicated by an asterisk. Bootstrap support for the other nodes within the radiations range from 59.8% to 86.2% (not indicated). Branch lengths reflect the amount of genetic drift. Trees were rooted with *A*. *citrinellus* from L. Nicaragua. The direction of gene flow is indicated by arrows and heat colors reflect intensity. B) D) F) H) and J) Heat maps of the residual covariance among population pairs given the respective trees assuming no or up to four migration events. Pairwise comparisons between species from the crater lakes and the source lakes are highlighted in a black box. Note that heat maps are differently scaled. Given above each covariance matrix is the fraction of variance explained in the observed covariance matrix by the tree (“f”).
(TIF)

S8 Fig Phylogenetic networks. Neighbor-net phylogenetic split networks based on uncorrected P-distances calculated from 15,780 and 16,542 SNPs for A) L. Apoyo and B) L. Xiloá, respectively.
(TIF)

S9 Fig One- and two-population models. Schematic illustration of A) one-population and B) two-population models. Note that growth was modelled to be exponential and not linear as depicted here. Migration rates are indicated in forward time.
(TIF)

S1 Table Sample sizes and composition of species / genetic clusters. (DOCX)

S2 Table Overall pairwise genetic differentiation. (DOCX)

S3 Table Outlier loci detected in global and pairwise analyses with BayeScan and the FLK test in L. Apoyo and L. Xiloá. (XLSX)

S4 Table Support for one-population models defined in S9A Fig. (DOCX)

S5 Table Support for two-population models defined in S9B Fig. (DOCX)

S6 Table Divergence times that happened before bottleneck can be inferred correctly according to simulation. (DOCX)

S7 Table Individual- and population-level information on read number, coverage, genomic library identity, and missing data. (XLSX)

S1 Text Genomic differentiation and signatures of selection during repeated sympatric speciation. (DOCX)

S1 Dataset Data files for all population genomic and phylogenetic analyses as well as site frequency spectra. (ZIP)
