# Supplementary figures and images for: Multispecies Outcomes of Sympatric Speciation after Admixture with the Source Population in Two Radiations of Nicaraguan Crater Lake Cichlids
Source: PLoS Genet. 2016 Jun 30;12(6):e1006157. doi: 10.1371/journal.pgen.1006157 (PMC4928843; doi:10.1371/journal.pgen.1006157)

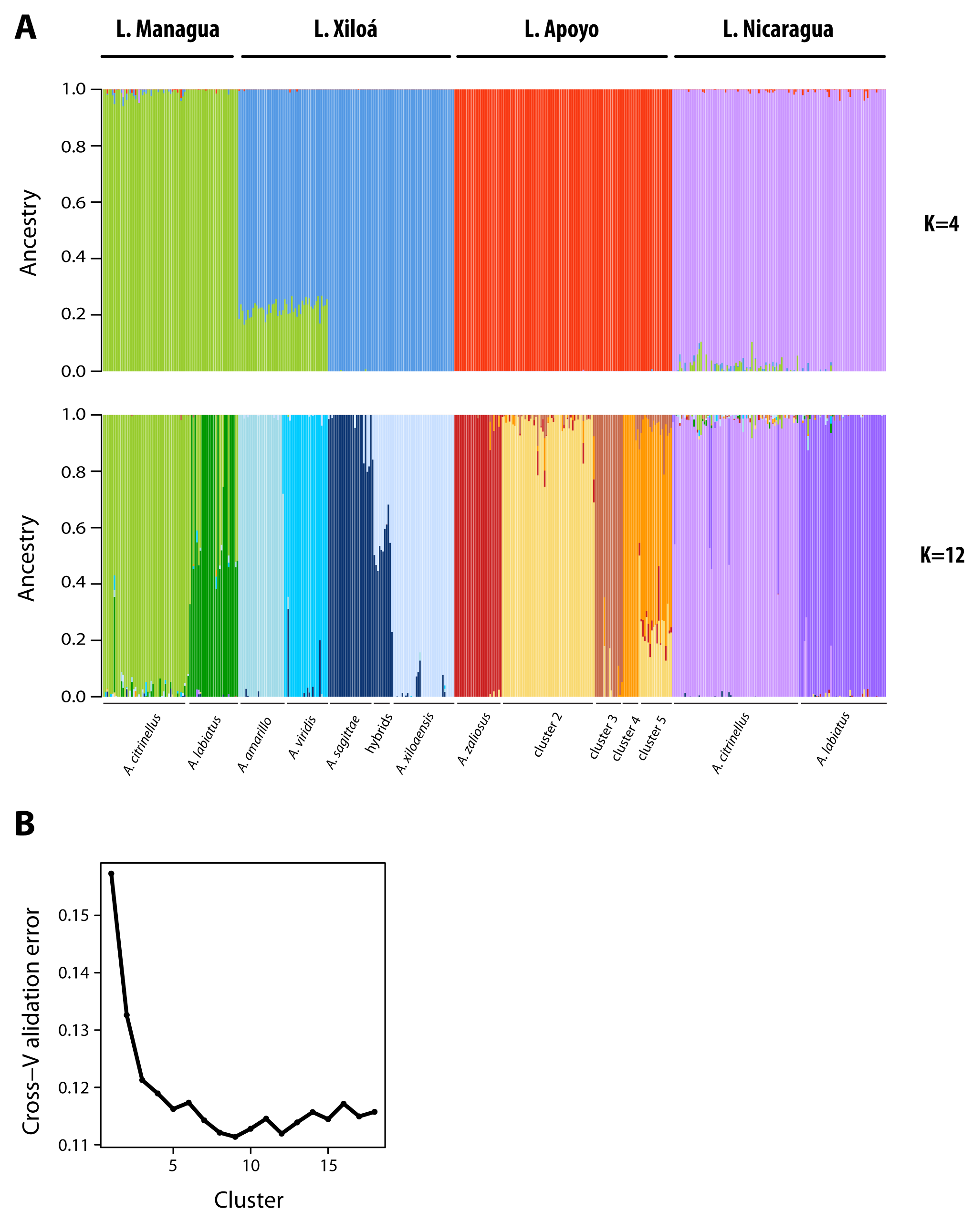

Supplement: S1 Fig — A) Shown are the result assuming the same number of clusters as lakes (K = 4) and the most supported number of clusters (K = 12). Note that K = 9 is equally well supported, but results for K = 12 are shown as this more closely reflects the number of populations in our data set. B) Support for all runs assuming a priori 1–18 clusters. A lower cross-validation error indicates higher support. (TIF) [file pgen.1006157.s001.tif]

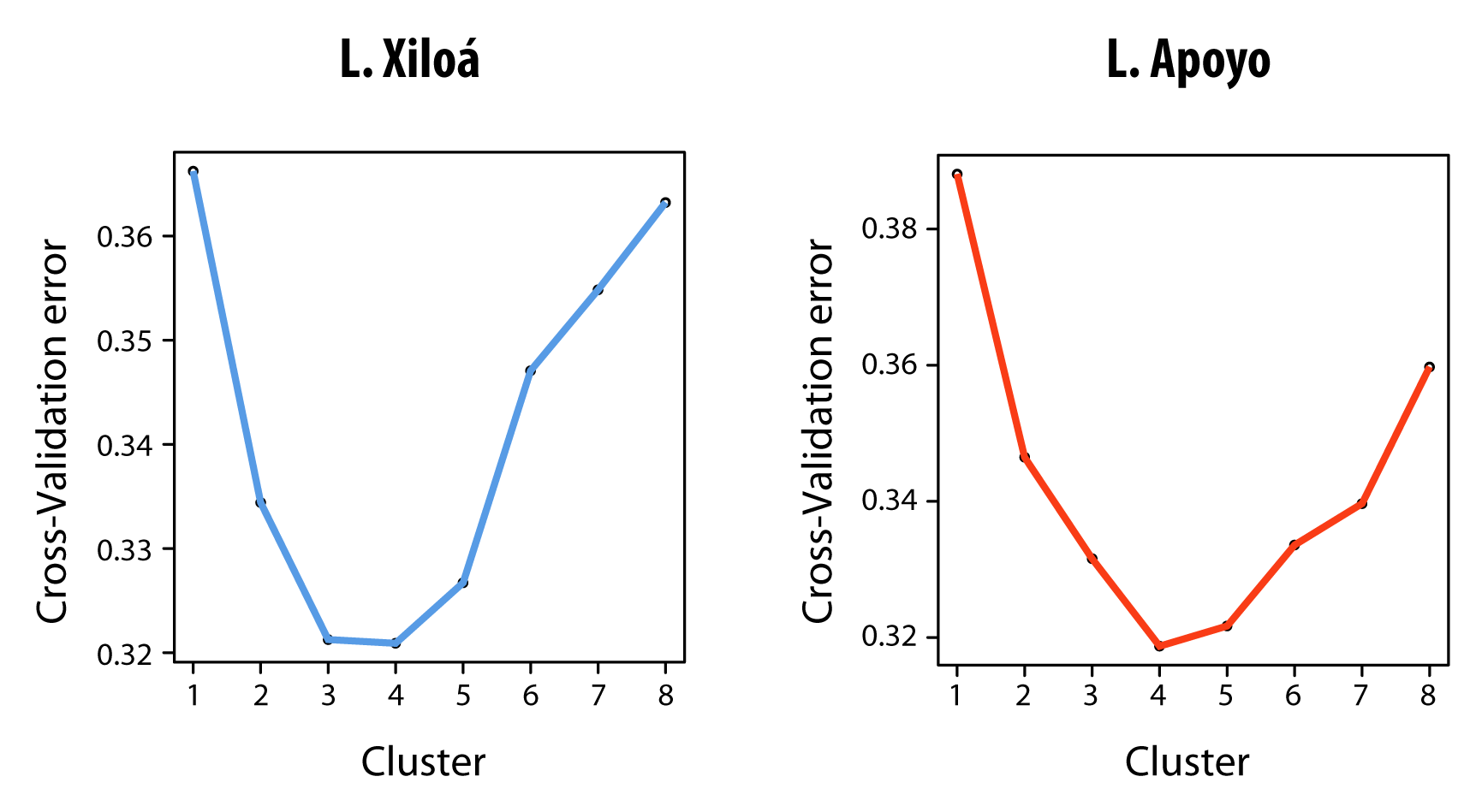

Supplement: S2 Fig — For both crater lake radiations between 1–8 different clusters were assumed a priori. A lower cross-validation error indicates higher support. (TIF) [file pgen.1006157.s002.tif]

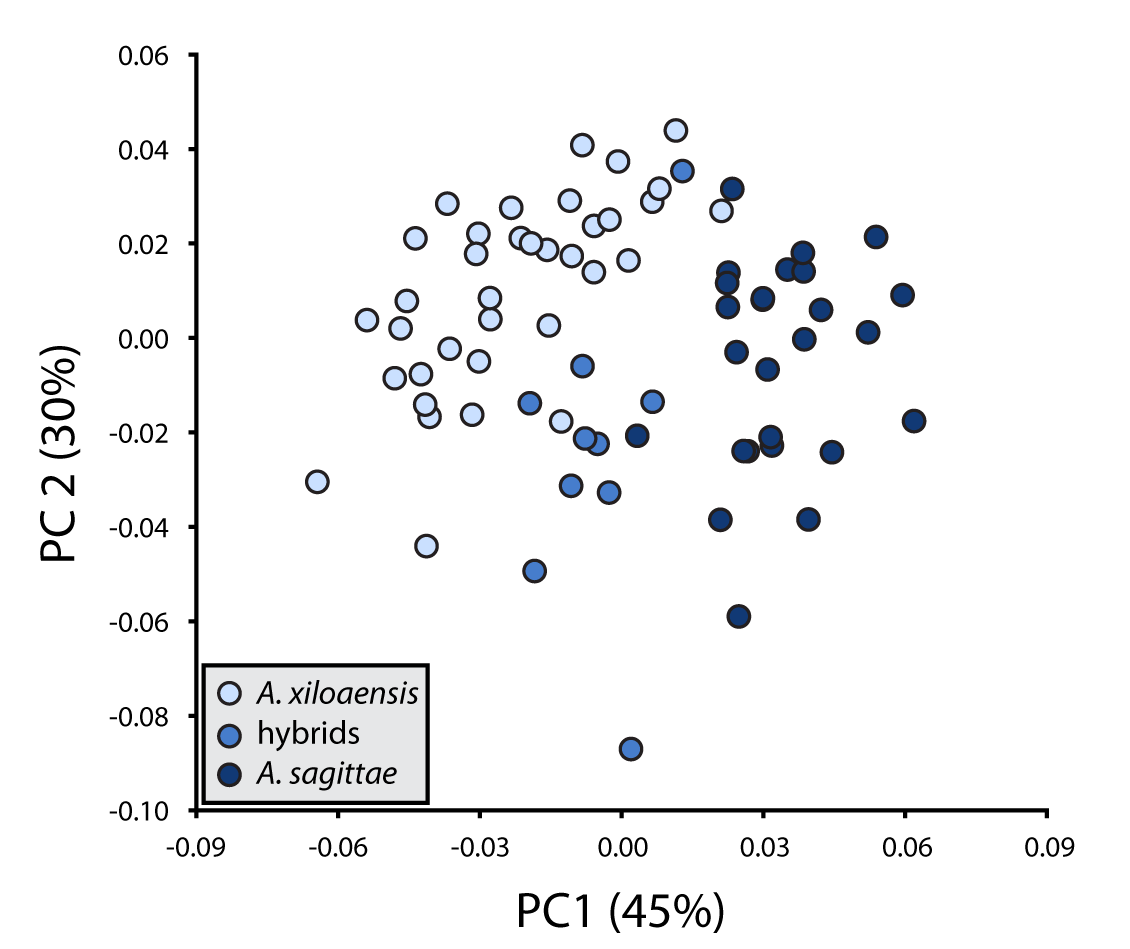

Supplement: S3 Fig — Shown are the first two axes of a principal component analysis based on seven homologous landmarks. Individuals genetically assigned to the hybrid group in L. Xiloá occupy an intermediate position between the limnetic species A. sagittae and the benthic species A. xiloaensis. (TIF) [file pgen.1006157.s003.tif]

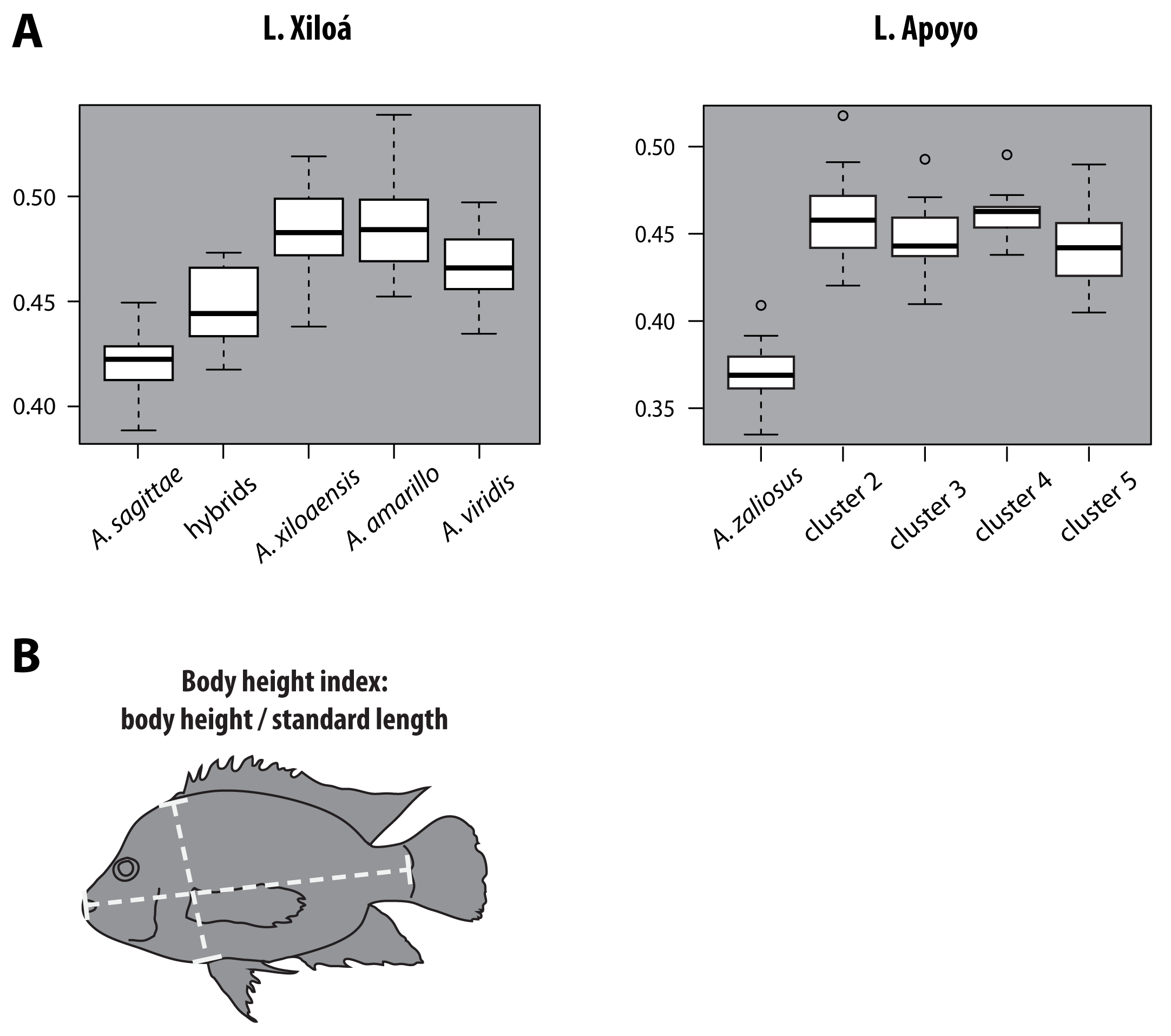

Supplement: S4 Fig — A) In L. Xiloá the limnetic species A. sagittae exhibits the most elongated body shape (lower BHI). The hybrid group is intermediate to A. sagittae and the rest of the benthic species. In L. Apoyo the limnetic A. zaliosus is markedly more elongated than the other four benthic species. B) Schematic illustration of the BHI. (TIF) [file pgen.1006157.s004.tif]

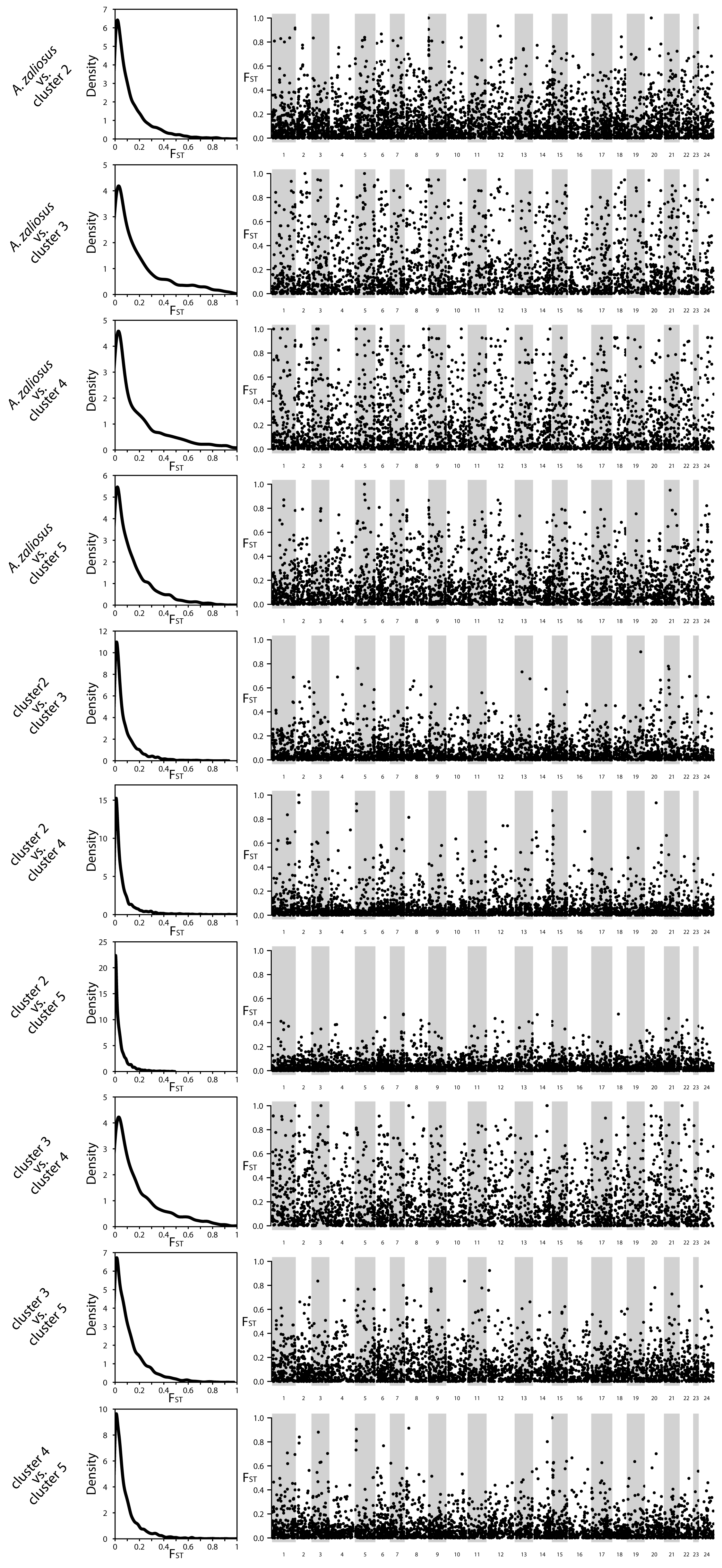

Supplement: S5 Fig — For all ten pairwise comparisons the overall distribution of FST-values (density plot) and the genetic differentiation across the 24 linkage groups of the reference genome are given. Markers with a minor allele frequency (MAF) below 5% were excluded. The number of markers for each comparison is given in S3 Table. (TIF) [file pgen.1006157.s005.tif]

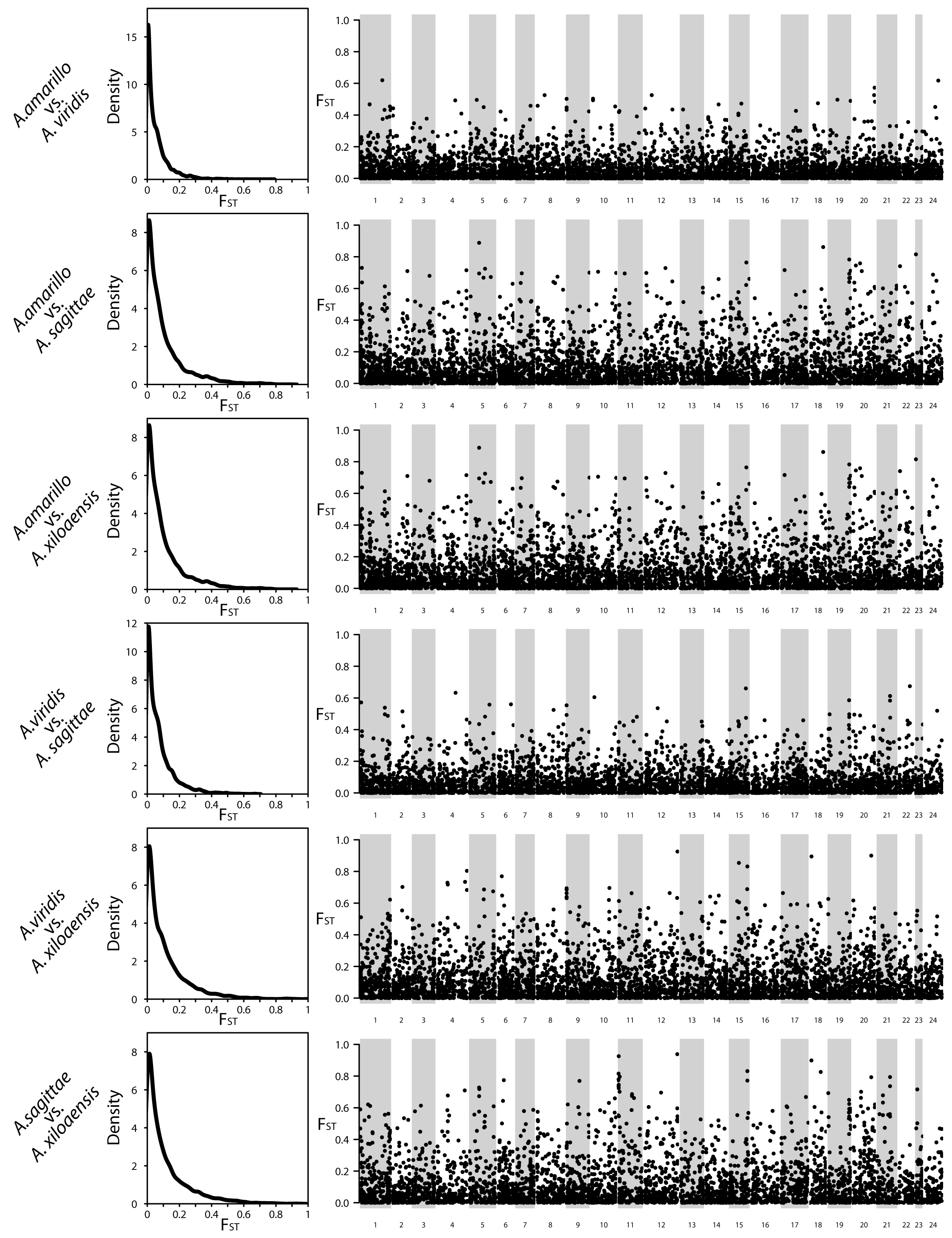

Supplement: S6 Fig — For all six pairwise comparisons the overall distribution of FST-values (density plot) and the genetic differentiation across the 24 linkage groups of the reference genome are given. Markers with a minor allele frequency (MAF) below 5% were excluded. The number of markers for each comparison is given in S3 Table. (TIF) [file pgen.1006157.s006.tif]

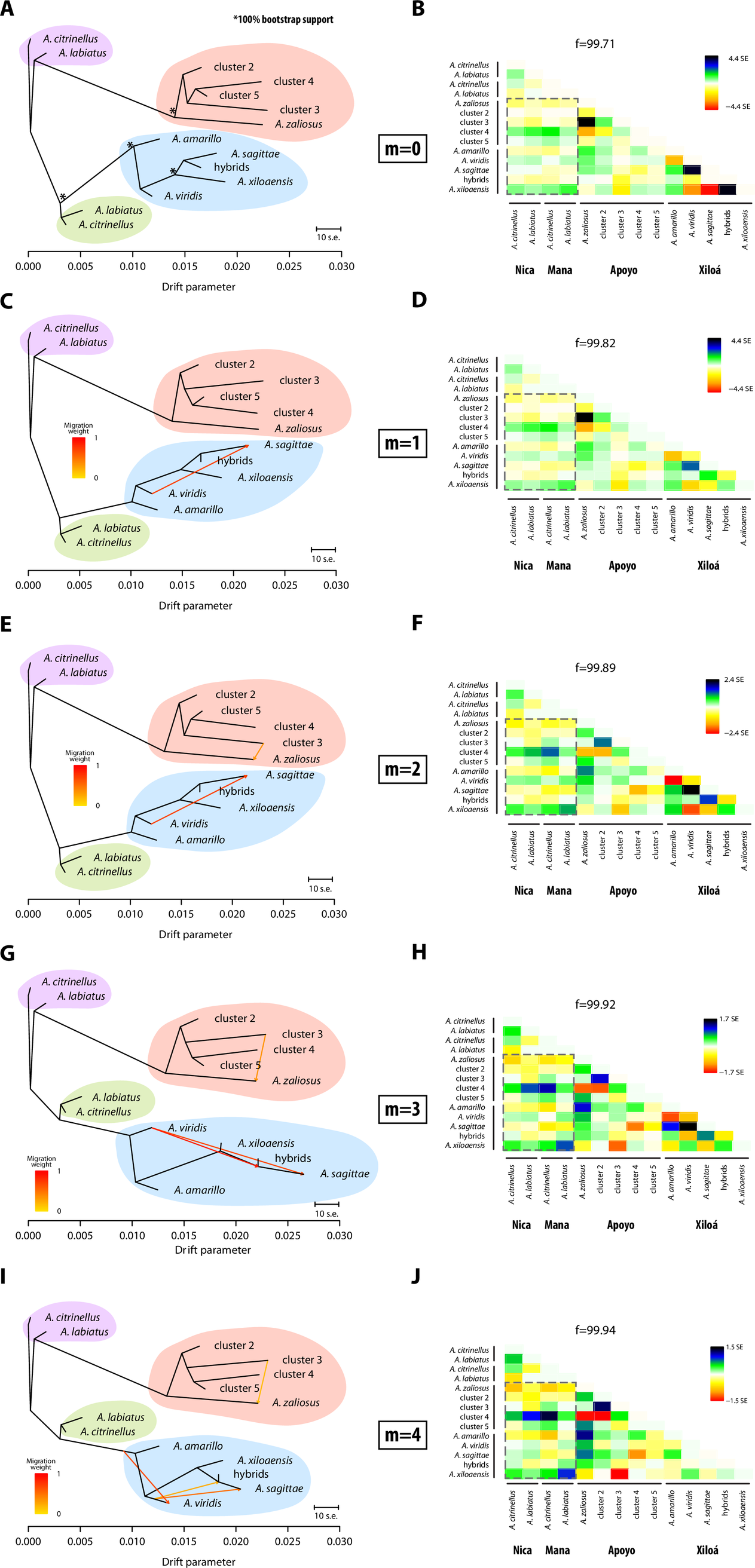

Supplement: S7 Fig — Maximum likelihood trees based on allele frequencies at 13,477 loci located on the 24 linkage groups with A) no migration (m = 0) or C) E) G) I) fitting up to four migration events (m = 1–4) on the tree. Lakes are indicated by color-shading. Nodes that have 100% bootstrap support are indicated by an asterisk. Bootstrap support for the other nodes within the radiations range from 59.8% to 86.2% (not indicated). Branch lengths reflect the amount of genetic drift. Trees were rooted with A. citrinellus from L. Nicaragua. The direction of gene flow is indicated by arrows and heat colors reflect intensity. B) D) F) H) and J) Heat maps of the residual covariance among population pairs given the respective trees assuming no or up to four migration events. Pairwise comparisons between species from the crater lakes and the source lakes are highlighted in a black box. Note that heat maps are differently scaled. Given above each covariance matrix is the fraction of variance explained in the observed covariance matrix by the tree (“f”). (TIF) [file pgen.1006157.s007.tif]

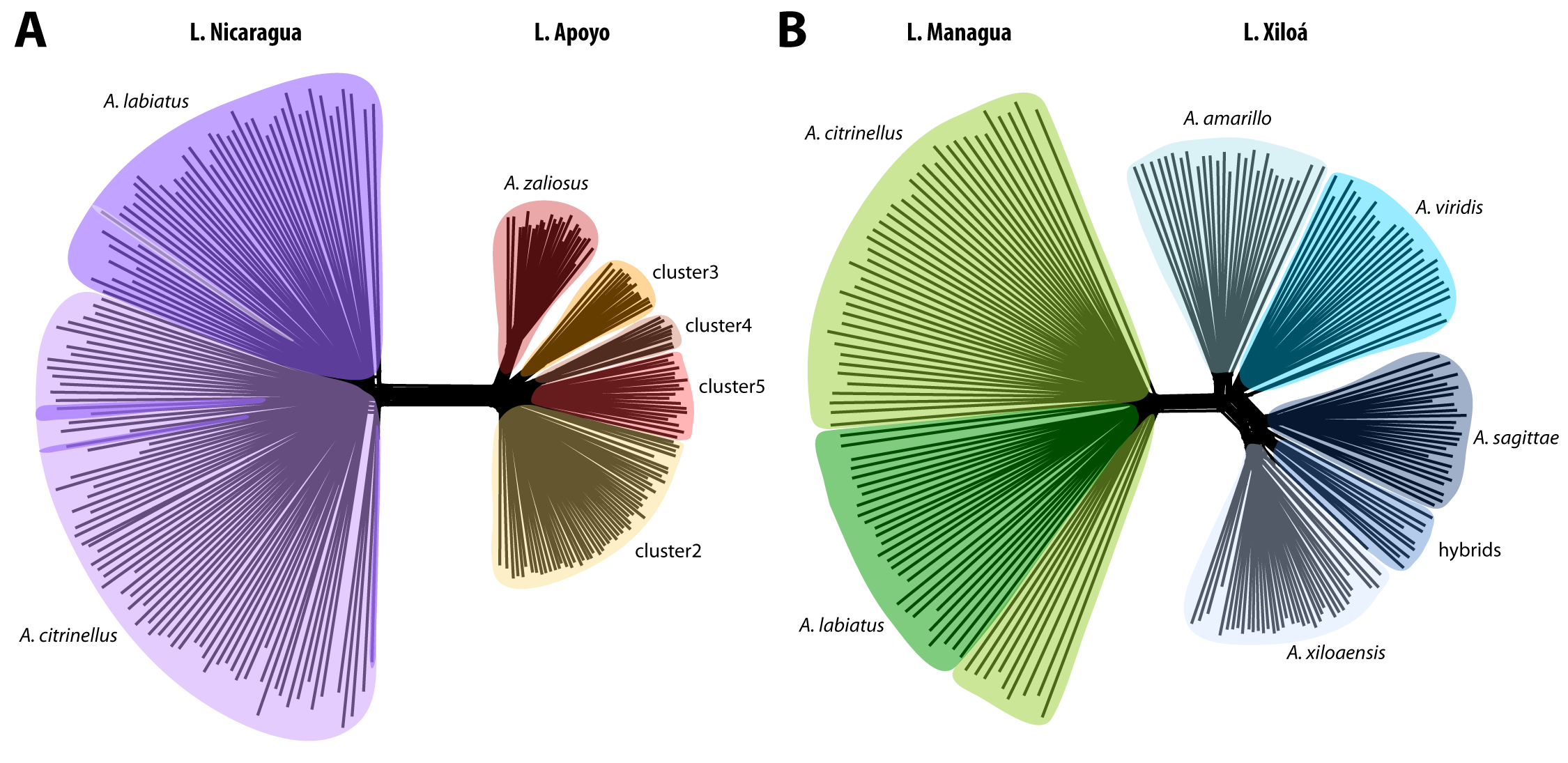

Supplement: S8 Fig — Neighbor-net phylogenetic split networks based on uncorrected P-distances calculated from 15,780 and 16,542 SNPs for A) L. Apoyo and B) L. Xiloá, respectively. (TIF) [file pgen.1006157.s008.tif]

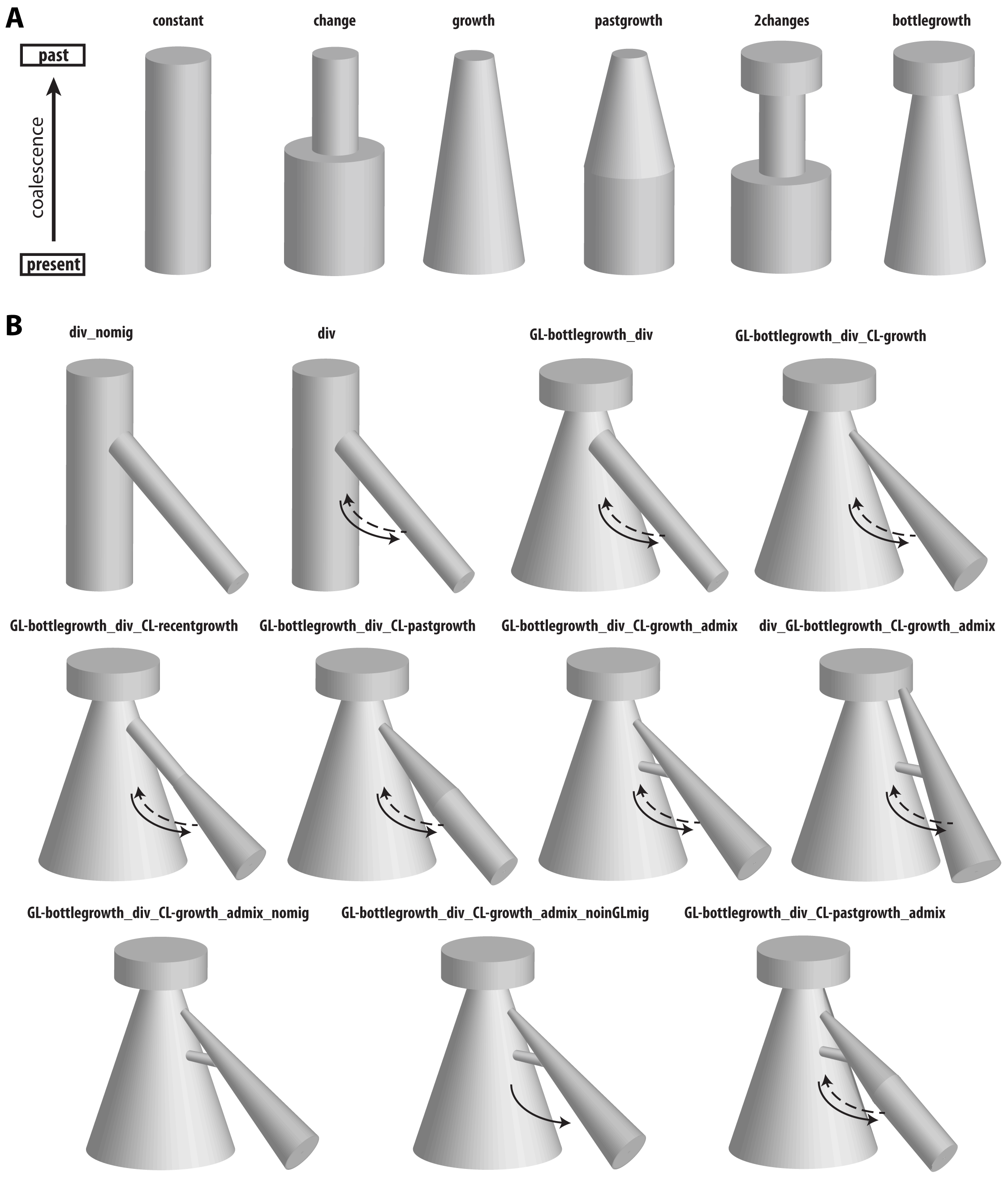

Supplement: S9 Fig — Schematic illustration of A) one-population and B) two-population models. Note that growth was modelled to be exponential and not linear as depicted here. Migration rates are indicated in forward time. (TIF) [file pgen.1006157.s009.tif]
