# Supplementary material for: Multispecies Outcomes of Sympatric Speciation after Admixture with the Source Population in Two Radiations of Nicaraguan Crater Lake Cichlids
Source: PLoS Genet. 2016 Jun 30;12(6):e1006157. doi: 10.1371/journal.pgen.1006157 (PMC4928843; doi:10.1371/journal.pgen.1006157)
Supplement: S1 Table — (DOCX) [file pgen.1006157.s010.docx]

**Table S1. Sample sizes and composition of species / genetic clusters.**

| **Lake** | **Species / genetic cluster** | **Sample size** | **Original species assignment** |
| --- | --- | --- | --- |
| L. Nicaragua | *A. citrinellus* | 72 |  |
|  | *A. labiatus* | 50 |  |
| L. Managua | *A. citrinellus* | 50 |  |
|  | *A. labiatus* | 27 |  |
| L. Apoyo | *A. zaliosus* | 27 |  |
|  | cluster 2 | 53 | *A. astorquii* (24), *A. flaveolus* (11), *A chancho* (9), *A. superciliu*s (8), *A. globosus* (1), |
|  | cluster 3 | 16 | *A. chancho* (9), *A. flaveolus* (7) |
|  | cluster 4 | 9 | *A. globosus* (8), *A. chancho* (1) |
|  | cluster 5 | 19 | *A. flaveolus* (7), *A. chancho* (5), *A. globosus* (4), *A. flaveolus* (3) |
| L. Xiloá | *A. amarillo* | 26 | *A. viridis* (3) |
|  | *A. viridis* | 25 |  |
|  | *A. sagittae* | 26 | *A. xiloaensis* (4) |
|  | hybrids | 10 | *A. xiloaensis* (8), *A. sagittae* (2) |
|  | *A. xiloaensis* | 36 |  |

The last column gives the composition of the different genetic clusters with number of individuals in parentheses.
