# Supplementary material for: Multispecies Outcomes of Sympatric Speciation after Admixture with the Source Population in Two Radiations of Nicaraguan Crater Lake Cichlids
Source: PLoS Genet. 2016 Jun 30;12(6):e1006157. doi: 10.1371/journal.pgen.1006157 (PMC4928843; doi:10.1371/journal.pgen.1006157)
Supplement: S2 Table — (DOCX) [file pgen.1006157.s011.docx]

**Table S2. Overall pairwise genetic differentiation.**

| **Lake** |  | **L. Managua** | | **L. Xiloá** | | | | | **L. Apoyo** | | | | | **L. Nicaragua** |
| --- | --- | --- | --- | --- | --- | --- | --- | --- | --- | --- | --- | --- | --- | --- |
|  | **Species** | ***A. citrinellus*** | ***A. labiatus*** | ***A. amarillo*** | ***A. viridis*** | ***A. sagittae*** | **hybrids** | ***A. xiloaensis*** | ***A. zaliosus*** | **cluster2** | **cluster3** | **cluster4** | **cluster5** | ***A. citrinellus*** |
| **L. Managua** | ***A. labiatus*** | 0.031 |  |  |  |  |  |  |  |  |  |  |  |  |
| **L. Xiloá** | ***A. amarillo*** | 0.148 | 0.183 |  |  |  |  |  |  |  |  |  |  |  |
|  | ***A. viridis*** | 0.121 | 0.152 | 0.104 |  |  |  |  |  |  |  |  |  |  |
|  | ***A. sagittae*** | 0.193 | 0.243 | 0.199 | 0.129 |  |  |  |  |  |  |  |  |  |
|  | **hybrids** | 0.172 | 0.217 | 0.189 | 0.135 | 0.109 |  |  |  |  |  |  |  |  |
|  | ***A. xiloaensis*** | 0.226 | 0.282 | 0.233 | 0.205 | 0.228 | 0.129 |  |  |  |  |  |  |  |
| **L. Apoyo** | ***A. zaliosus*** | 0.332 | 0.395 | 0.513 | 0.487 | 0.568 | 0.577 | 0.583 |  |  |  |  |  |  |
|  | **cluster2** | 0.300 | 0.349 | 0.448 | 0.427 | 0.489 | 0.484 | 0.514 | 0.242 |  |  |  |  |  |
|  | **cluster3** | 0.311 | 0.366 | 0.495 | 0.471 | 0.556 | 0.560 | 0.573 | 0.367 | 0.196 |  |  |  |  |
|  | **cluster4** | 0.283 | 0.336 | 0.466 | 0.437 | 0.538 | 0.531 | 0.555 | 0.379 | 0.198 | 0.314 |  |  |  |
|  | **cluster5** | 0.265 | 0.312 | 0.432 | 0.406 | 0.487 | 0.474 | 0.515 | 0.265 | 0.093 | 0.212 | 0.172 |  |  |
| **L. Nicaragua** | ***A. citrinellus*** | 0.050 | 0.073 | 0.169 | 0.147 | 0.207 | 0.189 | 0.242 | 0.286 | 0.254 | 0.266 | 0.247 | 0.223 |  |
|  | ***A. labiatus*** | 0.064 | 0.085 | 0.185 | 0.164 | 0.230 | 0.208 | 0.265 | 0.303 | 0.271 | 0.283 | 0.257 | 0.232 | 0.018 |

Pairwise F_ST_-values calculated from a matrix of 17,930 SNPs. All comparisons are highly significant (p< 1x 10^-4^).
