# Supplementary material for: Multispecies Outcomes of Sympatric Speciation after Admixture with the Source Population in Two Radiations of Nicaraguan Crater Lake Cichlids
Source: PLoS Genet. 2016 Jun 30;12(6):e1006157. doi: 10.1371/journal.pgen.1006157 (PMC4928843; doi:10.1371/journal.pgen.1006157)
Supplement: S4 Table — (DOCX) [file pgen.1006157.s013.docx]

**Table S4. Support for one-population models defined in Fig S9A.**

| **Lake** | **Species** | **SNPs / total sites^a^** | **Model** | **# fsc2 runs** | **# parameters** | **Ln-Lhood** | **ΔAIC** | **w_i_** |
| --- | --- | --- | --- | --- | --- | --- | --- | --- |
| Nicaragua | *A. citrinellus* | 12,879 / 4,229,008 | bottlegrowth | 100 | 4 | -122611.373 | - | 0.748 |
|  |  |  | 2changes | 50 | 5 | -122611.461 | 2.175 | 0.252 |
|  |  |  | change | 50 | 3 | -122636.865 | 48.984 | ~0 |
|  |  |  | growth | 50 | 3 | -122637.155 | 49.564 | ~0 |
|  |  |  | pastgrowth | 50 | 4 | -122637.213 | 51.679 | ~0 |
|  |  |  | constant | 50 | 1 | -122811.035 | 393.324 | ~0 |
|  | *A. labiatus* | 9,723 / 3,442,894 | bottlegrowth | 75 | 4 | -93907.015 | - | 0.567 |
|  |  |  | 2changes | 25 | 5 | -93906.283 | 0.536 | 0.433 |
|  |  |  | growth | 25 | 3 | -93921.945 | 27.860 | ~0 |
|  |  |  | change | 25 | 3 | -93921.949 | 27.869 | ~0 |
|  |  |  | pastgrowth | 25 | 4 | -93923.211 | 32.393 | ~0 |
|  |  |  | constant | 25 | 1 | -93965.815 | 111.602 | ~0 |
| Managua | *A. citrinellus* | 12,998 / 5,111,007 | bottlegrowth | 75 | 4 | -127745.092 | - | 0.717 |
|  |  |  | 2changes | 25 | 5 | -127745.023 | 1.862 | 0.283 |
|  |  |  | growth | 25 | 3 | -127880.100 | 268.015 | ~0 |
|  |  |  | change | 25 | 3 | -127880.687 | 269.189 | ~0 |
|  |  |  | pastgrowth | 25 | 4 | -127883.132 | 276.080 | ~0 |
|  |  |  | constant | 25 | 1 | -127908.092 | 320.000 | ~0 |
|  | *A. labiatus* | 10,833 / 5,061,764 | bottlegrowth | 75 | 4 | -104547.428 | - | 0.773 |
|  |  |  | 2changes | 25 | 5 | -104547.658 | 2.461 | 0.226 |
|  |  |  | growth | 25 | 3 | -104555.206 | 13.556 | 0.001 |
|  |  |  | change | 25 | 3 | -104554.815 | 14.773 | ~0 |
|  |  |  | pastgrowth | 25 | 4 | -104557.304 | 17.752 | ~0 |
|  |  |  | constant | 25 | 1 | -104626.352 | 151.847 | ~0 |
| Nicaragua | *A. citrinellus*  (>=15x coverage^b^) | 6,089 / 2,064,116 | bottlegrowth | 50 | 4 | -58310.216 | - | 0.563 |
|  |  |  | 2changes | 50 | 5 | -58309.468 | 0.503 | 0.437 |
|  |  |  | change | 50 | 3 | -58325.560 | 28.689 | ~0 |
|  |  |  | growth | 50 | 3 | -58325.581 | 28.730 | ~0 |
|  |  |  | pastgrowth | 50 | 4 | -58326.214 | 31.997 | ~0 |
|  |  |  | constant | 50 | 1 | -58390.850 | 155.268 | ~0 |

Given are the number of independent fastsimcoal2 runs, number of parameters, Ln-Likelihood, delta AIC and Akaike’s weight of evidence (w_i_**_)_**. The latter two are in reference to the best model for each species.

^a^ Number of segregating and total sites used to build the respective site frequency spectra.

^b^ Analyses for this species were repeated using only genotype calls based on a read depth of at least 15x.
