# Supplementary material for: Multispecies Outcomes of Sympatric Speciation after Admixture with the Source Population in Two Radiations of Nicaraguan Crater Lake Cichlids
Source: PLoS Genet. 2016 Jun 30;12(6):e1006157. doi: 10.1371/journal.pgen.1006157 (PMC4928843; doi:10.1371/journal.pgen.1006157)
Supplement: S5 Table — (DOCX) [file pgen.1006157.s014.docx]

**Table S5. Support for two-population models defined in Fig S9B.**

| **Lake** | **Species** | **SNPs / total sites^a^** | **Model** | **# fsc2 runs** | **# parameters** | **Ln-Lhood** | **ΔAIC** | **w_i_** |
| --- | --- | --- | --- | --- | --- | --- | --- | --- |
| Apoyo | *A. zaliosus* | 12,320 / 3,885,839 | GL-bottlegrowth_div_CL-growth_admix_nomig | 75 | 9 | -134914.336 | - | 0.592 |
|  |  |  | GL-bottlegrowth_div_CL-growth_admix | 25 | 11 | -134912.724 | 0.776 | 0.401 |
|  |  |  | div_GL-bottlegrowth_CL-growth_admix | 25 | 11 | -134916.749 | 8.826 | 0.007 |
|  |  |  | GL-bottlegrowth_div_CL-growth | 25 | 9 | -134930.809 | 32.945 | ≈ 0 |
|  |  |  | GL-bottlegrowth_div_CL-recentgrowth | 25 | 10 | -134930.942 | 35.212 | ≈ 0 |
|  |  |  | GL-bottlegrowth_div_CL-pastgrowth | 25 | 10 | -134931.743 | 36.815 | ≈ 0 |
|  |  |  | GL-bottlegrowth_div | 25 | 8 | -134999.854 | 169.036 | ≈ 0 |
|  |  |  | div | 25 | 5 | -135181.157 | 525.642 | ≈ 0 |
|  |  |  | div_nomig | 25 | 3 | -135493.372 | 1146.071 | ≈ 0 |
|  | cluster 2 | 12,273 / 3,794,781 | GL-bottlegrowth_div_CL-growth_admix_nomig | 75 | 9 | -136193.014 | - | 0.927 |
|  |  |  | GL-bottlegrowth_div_CL-growth_admix | 25 | 11 | -136193.549 | 5.068 | 0.073 |
|  |  |  | GL-bottlegrowth_div_CL-growth | 25 | 9 | -136209.192 | 32.356 | ≈ 0 |
|  |  |  | div_GL-bottlegrowth_CL-growth_admix | 25 | 11 | -136207.889 | 33.749 | ≈ 0 |
|  |  |  | GL-bottlegrowth_div_CL-recentgrowth | 25 | 10 | -136210.222 | 36.414 | ≈ 0 |
|  |  |  | GL-bottlegrowth_div_CL-pastgrowth | 25 | 10 | -136211.106 | 38.183 | ≈ 0 |
|  |  |  | GL-bottlegrowth_div | 25 | 8 | -136382.218 | 376.407 | ≈ 0 |
|  |  |  | div | 25 | 5 | -136565.994 | 737.959 | ≈ 0 |
|  |  |  | div_nomig | 25 | 3 | -136763.284 | 1128.539 | ≈ 0 |
|  | cluster 3 | 9,221 / 2,907,256 | GL-bottlegrowth_div_CL-growth_admix_nomig | 75 | 9 | -100002.319 | - | 0.910 |
|  |  |  | GL-bottlegrowth_div_CL-growth_admix | 25 | 11 | -100002.634 | 4.631 | 0.090 |
|  |  |  | div_GL-bottlegrowth_CL-growth_admix | 25 | 11 | -100009.623 | 18.608 | ≈ 0 |
|  |  |  | GL-bottlegrowth_div_CL-growth | 25 | 9 | -100012.703 | 20.769 | ≈ 0 |
|  |  |  | GL-bottlegrowth_div_CL-pastgrowth | 25 | 10 | -100012.045 | 21.452 | ≈ 0 |
|  |  |  | GL-bottlegrowth_div_CL-recentgrowth | 25 | 10 | -100013.675 | 24.713 | ≈ 0 |
|  |  |  | GL-bottlegrowth_div | 25 | 8 | -100070.16 | 133.682 | ≈ 0 |
|  |  |  | div | 25 | 5 | -100193.394 | 374.151 | ≈ 0 |
|  |  |  | div_nomig | 25 | 3 | -100424.562 | 832.487 | ≈ 0 |
|  | cluster 4 | 8,586 / 2,741,040 | GL-bottlegrowth_div_CL-growth_admix_nomig | 75 | 9 | -92206.6548 | - | 0.601 |
|  |  |  | GL-bottlegrowth_div_CL-growth | 25 | 9 | -92207.6564 | 2.003 | 0.221 |
|  |  |  | GL-bottlegrowth_div_CL-recentgrowth | 25 | 10 | -92207.5183 | 3.727 | 0.093 |
|  |  |  | GL-bottlegrowth_div_CL-pastgrowth | 25 | 10 | -92208.1354 | 4.961 | 0.050 |
|  |  |  | GL-bottlegrowth_div_CL-growth_admix | 25 | 11 | -92207.5229 | 5.736 | 0.034 |
|  |  |  | div_GL-bottlegrowth_CL-growth_admix | 25 | 11 | -92216.7171 | 24.125 | ≈ 0 |
|  |  |  | GL-bottlegrowth_div | 25 | 8 | -92232.5152 | 49.721 | ≈ 0 |
|  |  |  | div | 25 | 5 | -92366.3115 | 311.313 | ≈ 0 |
|  |  |  | div_nomig | 25 | 3 | -92455.1406 | 484.972 | ≈ 0 |
|  | cluster 5 | 10,577 / 3,286,892 | GL-bottlegrowth_div_CL-growth_admix_nomig | 75 | 9 | -116067.839 | - | 0.710 |
|  |  |  | GL-bottlegrowth_div_CL-growth_admix | 25 | 11 | -116067.017 | 2.356 | 0.219 |
|  |  |  | GL-bottlegrowth_div_CL-growth | 25 | 9 | -116070.234 | 4.789 | 0.065 |
|  |  |  | GL-bottlegrowth_div_CL-recentgrowth | 25 | 10 | -116072.301 | 10.925 | 0.003 |
|  |  |  | GL-bottlegrowth_div_CL-pastgrowth | 25 | 10 | -116072.35 | 11.022 | 0.003 |
|  |  |  | div_GL-bottlegrowth_CL-growth_admix | 25 | 11 | -116075.203 | 18.727 | ≈ 0 |
|  |  |  | GL-bottlegrowth_div | 25 | 8 | -116162.146 | 186.614 | ≈ 0 |
|  |  |  | div | 25 | 5 | -116319.993 | 496.308 | ≈ 0 |
|  |  |  | div_nomig | 25 | 3 | -116441.565 | 735.451 | ≈ 0 |
|  | cluster 2  (>= 15x coverage^a^) | 4,360 / 1,372,640 | GL-bottlegrowth_div_CL-growth_admix_nomig | 75 | 9 | -48706.0993 | - | 0.926 |
|  |  |  | GL-bottlegrowth_div_CL-growth | 25 | 9 | -48708.7979 | 5.397 | 0.062 |
|  |  |  | GL-bottlegrowth_div_CL-growth_admix | 25 | 11 | -48708.8048 | 9.411 | 0.008 |
|  |  |  | div_GL-bottlegrowth_CL-growth_admix | 25 | 11 | -48709.7143 | 11.230 | 0.003 |
|  |  |  | GL-bottlegrowth_div | 25 | 8 | -48737.1842 | 60.170 | ≈ 0 |
|  |  |  | div | 25 | 5 | -48796.9385 | 173.679 | ≈ 0 |
|  |  |  | div_nomig | 25 | 3 | -48841.0699 | 257.941 | ≈ 0 |
| *Xiloá* | *A. amarillo* | 11,479 / 4,211,882 | GL-bottlegrowth_div_CL-growth_admix_noinGLmig | 75 | 10 | -135079.383 | - | 0.513 |
|  |  |  | GL-bottlegrowth_div_CL-growth_admix | 50 | 11 | -135078.522 | 0.278 | 0.446 |
|  |  |  | GL-bottlegrowth_div_CL-growth_admix_nomig | 50 | 9 | -135082.975 | 5.184 | 0.038 |
|  |  |  | div_GL-bottlegrowth_CL-growth_admix | 25 | 11 | -135083.638 | 10.510 | 0.003 |
|  |  |  | GL-bottlegrowth_div_CL-growth | 25 | 9 | -135093.721 | 26.676 | ≈ 0 |
|  |  |  | GL-bottlegrowth_div_CL-pastgrowth | 25 | 10 | -135094.009 | 29.252 | ≈ 0 |
|  |  |  | GL-bottlegrowth_div_CL-recentgrowth | 25 | 10 | -135095.676 | 32.586 | ≈ 0 |
|  |  |  | GL-bottlegrowth_div | 25 | 8 | -135299.542 | 436.319 | ≈ 0 |
|  |  |  | div | 25 | 5 | -135452.314 | 735.863 | ≈ 0 |
|  |  |  | div_nomig | 25 | 3 | -135816.473 | 1460.179 | ≈ 0 |
|  | *A. viridis* | 9,378 / 3,402,833 | GL-bottlegrowth_div_CL-growth_admix | 50 | 11 | -110511.777 | - | 0.543 |
|  |  |  | GL-bottlegrowth_div_CL-growth_admix_noinGLmig | 75 | 10 | -110513.495 | 1.435 | 0.265 |
|  |  |  | div_GL-bottlegrowth_CL-growth_admix | 25 | 11 | -110513.083 | 2.611 | 0.147 |
|  |  |  | GL-bottlegrowth_div_CL-growth_admix_nomig | 50 | 9 | -110516.286 | 5.017 | 0.044 |
|  |  |  | GL-bottlegrowth_div_CL-growth | 25 | 9 | -110521.227 | 14.900 | ≈ 0 |
|  |  |  | GL-bottlegrowth_div_CL-pastgrowth | 25 | 10 | -110520.423 | 15.292 | ≈ 0 |
|  |  |  | GL-bottlegrowth_div_CL-recentgrowth | 25 | 10 | -110520.898 | 16.241 | ≈ 0 |
|  |  |  | GL-bottlegrowth_div | 25 | 8 | -110629.506 | 229.458 | ≈ 0 |
|  |  |  | div | 25 | 5 | -110760.503 | 485.450 | ≈ 0 |
|  |  |  | div_nomig | 25 | 3 | -110969.416 | 899.278 | ≈ 0 |
|  | *A. sagittae* | 8,203 / 3,049,413 | GL-bottlegrowth_div_CL-growth_admix_noinGLmig | 75 | 10 | -95515.9968 | - | 0.767 |
|  |  |  | GL-bottlegrowth_div_CL-growth_admix | 50 | 11 | -95516.2662 | 2.539 | 0.215 |
|  |  |  | GL-bottlegrowth_div_CL-growth_admix_nomig | 50 | 9 | -95520.9128 | 7.832 | 0.015 |
|  |  |  | div_GL-bottlegrowth_CL-growth_admix | 25 | 11 | -95520.7378 | 11.482 | 0.002 |
|  |  |  | GL-bottlegrowth_div_CL-pastgrowth_admix | 25 | 12 | -95535.9302 | 43.867 | ≈ 0 |
|  |  |  | GL-bottlegrowth_div_CL-pastgrowth | 25 | 10 | -95545.1774 | 58.361 | ≈ 0 |
|  |  |  | GL-bottlegrowth_div_CL-growth | 25 | 9 | -95548.5714 | 63.149 | ≈ 0 |
|  |  |  | GL-bottlegrowth_div_CL-recentgrowth | 25 | 10 | -95548.5438 | 65.094 | ≈ 0 |
|  |  |  | GL-bottlegrowth_div | 25 | 8 | -95659.6781 | 283.363 | ≈ 0 |
|  |  |  | div | 25 | 5 | -95749.2256 | 456.458 | ≈ 0 |
|  |  |  | div_nomig | 25 | 3 | -96038.5132 | 1031.033 | ≈ 0 |
|  | *A. xiloaensis* | 11,676 / 4,347,260 | GL-bottlegrowth_div_CL-growth_admix_noinGLmig | 75 | 10 | -135680.892 | - | 0.888 |
|  |  |  | GL-bottlegrowth_div_CL-growth_admix | 50 | 11 | -135681.96 | 4.137 | 0.112 |
|  |  |  | GL-bottlegrowth_div_CL-growth_admix_nomig | 50 | 9 | -135690.986 | 18.189 | ≈ 0 |
|  |  |  | GL-bottlegrowth_div_CL-pastgrowth_admix | 25 | 12 | -135688.124 | 18.465 | ≈ 0 |
|  |  |  | div_GL-bottlegrowth_CL-growth_admix | 25 | 11 | -135691.999 | 24.215 | ≈ 0 |
|  |  |  | GL-bottlegrowth_div_CL-pastgrowth | 25 | 10 | -135700.648 | 39.512 | ≈ 0 |
|  |  |  | GL-bottlegrowth_div_CL-recentgrowth | 25 | 10 | -135709.884 | 57.984 | ≈ 0 |
|  |  |  | GL-bottlegrowth_div_CL-growth | 25 | 9 | -135712.585 | 61.386 | ≈ 0 |
|  |  |  | GL-bottlegrowth_div | 25 | 8 | -135828.255 | 290.726 | ≈ 0 |
|  |  |  | div | 25 | 5 | -135965.213 | 558.642 | ≈ 0 |
|  |  |  | div_nomig | 25 | 3 | -136324.874 | 1273.965 | ≈ 0 |

Given are the number of independent fastsimcoal2 runs, number of parameters, Ln-Likelihood, delta AIC, and Akaike’s weight of evidence (w_i_). The latter two are in reference to the best model for each species. Species from L. Apoyo were analyzed together with *A citrinellus* from L. Nicaragua, and species from L. Xiloá with *A. citrinellus* from L. Managua.

^a^ Number of segregating and total sites used to build the respective site frequency spectra.

^b^ Analyses for this species were repeated using only genotype calls based on a read depth of at least 15x.
