# Supplementary material for: Multispecies Outcomes of Sympatric Speciation after Admixture with the Source Population in Two Radiations of Nicaraguan Crater Lake Cichlids
Source: PLoS Genet. 2016 Jun 30;12(6):e1006157. doi: 10.1371/journal.pgen.1006157 (PMC4928843; doi:10.1371/journal.pgen.1006157)
Supplement: S6 Table — (DOCX) [file pgen.1006157.s015.docx]

**Table S6. Divergence times that happened before bottleneck can be inferred correctly according to simulation.**

|  | **Nanc** | **Nbot** | **Ncit** | **Nfounder** | **Ncl2** | **Tdiv** | **Tbot** | **Rcit** | **Rcl2** |
| --- | --- | --- | --- | --- | --- | --- | --- | --- | --- |
| **MLE** | 21,173 | 3,470 | 947,105 | 213 | 27,621 | 1,499 | 2,170 | -2.58 x 10^-3^ | -3.25 x 10^-3^ |
| **changed** |  |  |  |  |  | 11,499 |  |  | -4.23 x 10^-4^ |
| **inferred** | 21,240 | 3,997 | 362,794 | 213 | 27,606 | 11,440 | 2,476 | -1.82 x 10^-3^ | -4.25 x 10^-4^ |

Data was simulated according to maximum likelihood estimates (MLE) of a two-population model of cluster 2 in L. Apoyo, but 10,000 generations were added to the divergence time (changed). Note that the growth rate was adjusted accordingly (Rcl2). Parameters are defined as in the five-population model of Fig 4. There is some deviation in the timing of the bottleneck (Tbot) and the current size of the source population (Ncit), but importantly the divergence time (Tdiv) is inferred correctly.
