# Supplementary material for: Multispecies Outcomes of Sympatric Speciation after Admixture with the Source Population in Two Radiations of Nicaraguan Crater Lake Cichlids
Source: PLoS Genet. 2016 Jun 30;12(6):e1006157. doi: 10.1371/journal.pgen.1006157 (PMC4928843; doi:10.1371/journal.pgen.1006157)
Supplement: S1 Text — (DOCX) [file pgen.1006157.s017.docx]

**Genomic differentiation and signatures of selection during repeated sympatric speciation**

Since our data suggest that speciation happened in sympatry and we find evidence for ongoing gene flow among the sympatric species we wanted to examine the overall pattern of genetic differentiation across the genome and identify regions putatively under selection [1-4]. Furthermore, given the parallel evolution of limnetic and benthic species [5, 6] and the recent shared ancestry of the two radiations we were interested in identifying regions putatively under selection in both of the radiations. Pairwise differentiation across the genome was analyzed in terms of AMOVA F_ST_-values [7] as implemented in *Stacks* [8, 9]*.* In all pairwise comparisons of sympatric speciation genetic differentiation was heterogeneous across the genome, with most markers exhibiting relatively low levels and only few markers exhibiting high levels of differentiation (S8 and S9 Figs).

To identify regions in the genome putatively under selection we used *BayeScan* *v.2.1* [10] and the *FLK-test* [11]. Only loci for which at least ten individuals per species were successfully genotyped were used in these analyses, with the exception of cluster 4 in Apoyo for which the limit was set to six individuals due to its small sample size. *BayeScan* was run for all pairwise combinations of species in both crater lakes and for each crater lake in a global analysis including all species using the default settings. Global analyses have more power [12], but more markers can be retained in the pairwise analyses, since the chance that a locus will be excluded due to too much missing data in a population increases with the number of populations. Uninformative sites were excluded by applying a minor allele frequency (MAF) filter of 5 % [13]. While *BayeScan* has been shown to perform well under different demographic scenarios [14] the *FLK-test* may often be superior by taking the historical branching patterns and heterogeneity of genetic drift into account [15]. For both crater lakes species were analyzed jointly in a single analysis using the respective source population as outgroup. The population kinship matrix was estimated from our SNP data and the neutral distribution of the test statistic was obtained from 100,000 simulations performed with *simuPOP v.1.1.6* [16, 17]. No MAF filter was used for the *FLK-tests* as conditioning on intermediate allele frequencies can decrease the power of the test in small populations [11]. Outlier status in *BayeScan* was determined at a false discovery rate (FDR) of 5 %. In the case of *FLK-tests* markers were considered outliers if they exceeded the 99.5 percentile of the neutral distribution after fitting a smooth spline function [11].

Since we were concerned that the lower threshold of genotyped individuals of cluster 4 from L. Apoyo (minimum of six instead of ten individuals) might bias the results we repeated the analyses excluding it. Indeed, the number of outliers in the *FLK-test* dropped from 36 to only 20 after excluding cluster 4 and the 20 outliers are not merely a subset of the 36 but mostly different ones (S7 Table). Only five markers are shared in the two analyses. *BayeScan* on the other hand did not seem to be strongly affected. Upon removal of cluster 4 the number of outliers dropped from 22 to 18 in the global analyses; 15 of which are shared. We report the results from both analyses (S7 Table), but note that outliers found in analyses with cluster 4 only have to be considered with caution. Overall (including global and pairwise *BayeScan* analyses and *FLK-tests*) we found 126 outliers in L. Apoyo. Of these 93 were identified with *BayeScan*, 51 with *FLK-tests*, and 18 with both analyses. In L. Xiloá, overall a similar number (130) of outliers were detected, 100 with *BayeScan*, 54 with the *FLK-test*, and 24 with both (S8 Table). The proportion of outlier loci in pairwise or global analyses was relatively low with a range of 0 to 0.63 % in L. Apoyo and 0.11 to 0.74 % in L. Xiloá (S7 and S8 Tables), respectively. But note that the presence and number of outlier markers cannot be readily compared across the analyses as different subsets of SNPs were included.

In both radiations outlier markers are distributed among almost all of the 24 linkage groups (LG) and several unplaced scaffolds. But, notably, several outliers seem to occur in clusters. For example, out of the 21 outliers in the comparison of *A. zaliosus* versus cluster 2, eight were located within less than 500 kb on LG09 and five out of 16 outliers in the comparison of *A. amarillo* versus *A. viridis* resided within only 40 kb on scaffold UNK0036. Note that in the latter case two SNPs stemmed from the same RAD locus and were thus conservatively counted as one.

Interestingly, two SNP markers, ‘LG09 – 478,549’ and ‘UNK0899 – 17,760’, were found to be outliers in both radiations. Yet, neither of the two was consistently associated with high differentiation between the limnetic and benthic species. The former was found between the limnetic *A. zaliosus* and two of the benthic species in L. Apoyo, yet it was only an outlier in the two global analyses (*BayeScan* and *FLK-test*) in L. Xiloá and closer inspection revealed that it was caused by a high genetic differentiation between the two benthic species *A. amarillo* and *A. viridis* compared to the limnetic *A. sagittae* and the benthic *A. xiloaensis*. Marker ‘UNK0899 - 17,760’ was an outlier only in the global *FLK-test* in L. Apoyo and in two pairwise comparisons of benthic species (*A. amarillo* *and A. viridis* versus *A. xiloaensis*). Thus, while these two markers reside in genomic regions that may have been under selection in both radiations, they do not seem to have been involved in the parallel phenotypic evolution of limnetic and benthic species.

**References:**

1. Feder JL, Egan SP, Nosil P. The genomics of speciation-with-gene-flow. Trends Genet. 2012;28(7):342-50.

2. Feder JL, Flaxman SM, Egan SP, A. CA, Nosil P. Geographic Mode of Speciation and Genomic Divergence. Annu Rev Ecol Evol S. 2013;44:73-97.

3. Nosil P, Feder JL. Genomic divergence during speciation: causes and consequences. Philosophical Transactions of the Royal Society B. 2012;367(1587):332-42.

4. Nosil P, Funk DJ, Ortiz-Barrientos D. Divergent selection and heterogeneous genomic divergence. Mol Ecol. 2009;18(3):375-402.

5. Elmer KR, Fan SH, Kusche H, Spreitzer ML, Kautt AF, Franchini P, et al. Parallel evolution of Nicaraguan crater lake cichlid fishes via non-parallel routes. Nat Commun. 2014;5.

6. Kautt AF, Elmer KR, Meyer A. Genomic signatures of divergent selection and speciation patterns in a natural experiment', the young parallel radiations of Nicaraguan crater lake cichlid fishes. Mol Ecol. 2012;21(19):4770-86.

7. Weir BS, Cockerham CC. Estimating F-Statistics for the Analysis of Population-Structure. Evolution. 1984;38(6):1358-70.

8. Catchen J, Hohenlohe PA, Bassham S, Amores A, Cresko WA. Stacks: an analysis tool set for population genomics. Mol Ecol. 2013;22(11):3124-40.

9. Catchen JM, Amores A, Hohenlohe PA, Cresko WA, Postlethwait J. Stacks: building and genotyping loci *de novo* from short-read sequences. G3: Genes, Genomes, Genetics. 2011;1:171-82.

10. Foll M, Gaggiotti O. A Genome-Scan Method to Identify Selected Loci Appropriate for Both Dominant and Codominant Markers: A Bayesian Perspective. Genetics. 2008;180(2):977-93.

11. Bonhomme M, Chevalet C, Servin B, Boitard S, Abdallah J, Blott S, et al. Detecting Selection in Population Trees: The Lewontin and Krakauer Test Extended. Genetics. 2010;186(1):241-62.

12. Fischer MC, Foll M, Excoffier L, Heckel G. Enhanced AFLP genome scans detect local adaptation in high-altitude populations of a small rodent (*Microtus arvalis*). Mol Ecol. 2011;20(7):1450-62.

13. Roesti M, Salzburger W, Berner D. Uninformative polymorphisms bias genome scans for signatures of selection. BMC Evolutionary Biology. 2012;12.

14. Narum SR, Hess JE. Comparison of F-ST outlier tests for SNP loci under selection. Mol Ecol Resour. 2011;11:184-94.

15. Lotterhos KE, Whitlock MC. Evaluation of demographic history and neutral parameterization on the performance of F-ST outlier tests. Mol Ecol. 2014;23(9):2178-92.

16. Peng B, Amos CI. Forward-time simulations of non-random mating populations using simuPOP. Bioinformatics. 2008;24(11):1408-9.

17. Peng B, Kimmel M. simuPOP: a forward-time population genetics simulation environment. Bioinformatics. 2005;21(18):3686-7.
